# Research Progress in Epoxidation of Light Small-Molecule Olefins

**DOI:** 10.3390/molecules30061340

**Published:** 2025-03-17

**Authors:** Guanghui Zhao, Tianfu Yang, Jincheng Liu, Xianming Xu, Yulong Wang, Yongjun Zhang, Meng Gao, Chao Xiong, Hongbing Ji

**Affiliations:** 1 Daqing Petrochemical Research Center, PetroChina Petrochemical Research Institute, Da’qing 163714, China; 15645900343@163.com (G.Z.); liujinchengabc@126.com (J.L.); xianming_xu@126.com (X.X.); jlhd001@163.com (Y.W.); zhangyj459@petrochina.com.cn (Y.Z.); 15645967117@163.com (M.G.); 2 School of Chemistry and Chemical Engineering, Guangxi University, Nanning 530004, China; y978185171@163.com; 3 State Key Laboratory Breeding Base of Green-Chemical Synthesis Technology, Institute of Green Petroleum Processing and Light Hydrocarbon Conversion, College of Chemical Engineering, Zhejiang University of Technology, Hangzhou 310014, China

**Keywords:** light olefins, small molecules, epoxidation, catalysis mechanism, research progress

## Abstract

Light olefins, as important bulk raw materials in the petrochemical industry, play an irreplaceable role in the development of the manufacturing industry and the economy. The epoxides of light olefins are important intermediates for the synthesis of polymers, drugs, and fine chemicals, and their green, efficient, and safe synthesis has attracted much attention. This review focuses on the research progress of light olefin epoxidation and elucidates traditional epoxidation methods, such as the chlorohydrin method. Although these processes have mature processes, they have drawbacks, including equipment corrosion, environmental pollution, poor safety, and high waste emissions. Special emphasis is placed on catalytic epoxidation systems using oxygen or organic peroxides as oxygen sources. For homogeneous catalytic systems, certain metal complexes exhibit high activity and selectivity yet are difficult to separate and recycle. Moreover, heterogeneous catalytic systems have become a research hotspot due to their advantages of easy separation and reusability, with supported metal catalysts being a prime example. Meanwhile, the effects of reaction temperature, pressure, solvent, etc., on epoxidation are explored. The specific reaction mechanisms are also studied and analyzed. Current research challenges, including enhancing catalyst stability and reducing costs, are summarized. In the future, developing highly efficient, green, and economically viable epoxidation technologies for large-scale industrial applications represents an important research direction in this field.

## 1. Introduction

Olefin epoxidation reactions are a crucial class of reactions in the chemical industry, and they yield epoxides; these epoxides can serve as fundamental components of common chemicals and organic synthesis intermediates. Epoxides are among the most versatile and useful precursors in organic synthesis [1,2,3,4]. The production of ethylene oxide (EO), which can be used not only for the synthesis of ethylene glycol, ethylene glycol ethers, ethanolamines, and ethoxy ethers but also directly as a fumigant, bactericide, sterilant, or disinfectant, is one of the most crucial catalytic reactions that influence market value and chemical production volumes [5]. Propylene oxide (PO), which is a high-value-added commodity chemical, is used in the manufacture of a wide variety of products, among which the most important are polyurethane foams for the automotive and housing industries, polyester resins for the textile and construction industries, and propylene glycol that is used as a component in pharmaceuticals, cosmetics, and as an additive in heat-transfer or hydraulic fluids [6]. Butylene oxide (BO), a homolog of PO, is an important chemical intermediate that is widely used in the synthesis of organic intermediates and polymers, such as antioxidants, corrosion inhibitors, and surfactants [7].

Since silver does not break the C-H bond under oxidizing conditions, silver-based catalysts, which have been proven and applied in industry, have become one of the best catalysts for ethylene epoxidation. At low ethylene conversion rates (<10%), the EO selectivity of unpromoted and promoted Ag/α-Al_2_O_3_ catalysts is approximately 40–50% and 85–90%, respectively. From the perspectives of safety and environmental protection, high selectivity is crucial. It can prevent the release of a large amount of heat generated by the combustion of ethylene and EO, as well as the production of a certain quantity of CO_2_ [8,9]. Krishna R. Iyer et al. [10] investigated the impact of silver particle size on the ethylene epoxidation rate over Ag/α-Al_2_O_3_ catalysts with a particle size distribution ranging from 6.9 to 35 wt%. The epoxidation rates of these 16 samples were measured in the presence of an organic chloride promoter (3.5 ppm C_2_H_5_Cl). The results indicated that these samples exhibited nearly constant EO selectivity, approximately 75–81%. Among them, the optimal particle size distribution of Ag/α-Al_2_O_3_ centered around ~140 nm contributed to a high EO rate (>4 μmol/(g_Ag_·s)). However, the issue of low conversion rates still demands improvement, and enhancing selectivity remains of the utmost importance. Furthermore, compared with ethylene epoxidation, silver-based catalysts generally exhibit poor performance in propylene epoxidation. This is because the allylic hydrogen of propylene is highly susceptible to attack, leading to the complete oxidation of propylene [11]. Copper-based catalysts, which have been extensively studied as promising candidates for propylene epoxidation, have PO selectivity that has been reported to be unsatisfactory [12]. In addition, the selection of a process also exerts a certain degree of influence on the selectivity of PO. At present, in the industrial production of PO via propylene epoxidation, the primary methods include the chlorohydrin method, the co-oxidation method, and the hydrogen peroxide propylene oxide (HPPO) method. However, all three methods exhibit rather distinct disadvantages, namely that they may generate by-products detrimental to the environment, display low selectivity for PO, or necessitate costly liquid–liquid separation [13]. Meanwhile, BO is a by-product in the industrial preparation of PO via the chloropropane route. But this route suffers from severe equipment corrosion and generates a large amount of Cl^−^-containing wastewater [14]. Additionally, some researchers have studied the epoxidation of 1-butene over titanium silicalite-1 (TS-1), for which the catalytic activity and stability in the 1-butene epoxidation reaction are significantly lower than those in the propylene epoxidation reaction [7]. Therefore, a deep understanding of the catalytic mechanism is of great significance for the development of new methods or novel catalyst systems, aiming to significantly enhance the catalytic performance in the epoxidation of light olefins in this way.

This article reviews the main technological methods for the epoxidation of light olefins in recent years, introducing the background, advantages, and disadvantages of each process. It also summarizes some highly efficient catalysts used in the epoxidation of light olefins, elaborating in detail on the application of supported metal nano-catalysts among heterogeneous catalysts. Moreover, this review discusses the reaction mechanisms of the epoxidation of light olefins in different reaction systems, aiming to deeply analyze the specific reaction pathways and reveal the key factors influencing the reaction efficiency and selectivity. It is hoped that through these studies, a theoretical basis and technical support can be provided for the development of a more efficient and environmentally friendly epoxidation process for light olefins, thus promoting technological innovation and development in related fields.

## 2. Overview of Major Light Olefin Epoxidation Processes

**Figure 1:**
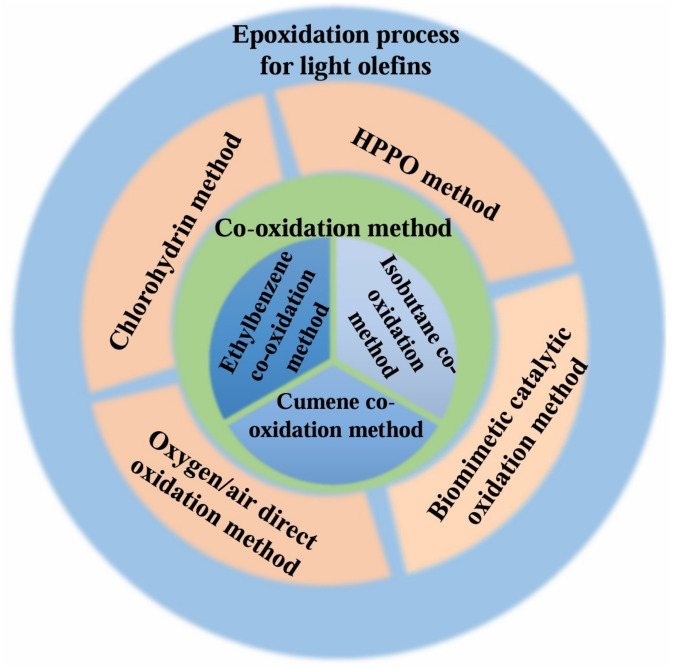
Main epoxidation processes for light olefins.

So far, the main processes for light olefin epoxidation that had been put into production or were under research included the chlorohydrin method [15,16,17,18], the hydrogen peroxide propylene oxide (HPPO) method [19,20,21,22,23,24,25,26,27,28,29,30,31,32,33,34,35,36,37], the oxygen/air direct oxidation method [38,39,40,41,42,43,44,45,46,47,48,49,50,51,52,53], the co-oxidation method [54,55,56,57,58,59,60,61,62,63,64,65,66,67,68,69,70,71,72,73,74,75,76], the biomimetic catalytic oxidation method [77,78,79,80,81,82,83,84,85,86,87,88], etc. As shown in Figure 1, the main process characteristics and research progress were as follows:

### 2.1. Chlorohydrin Method

**Figure 2:**
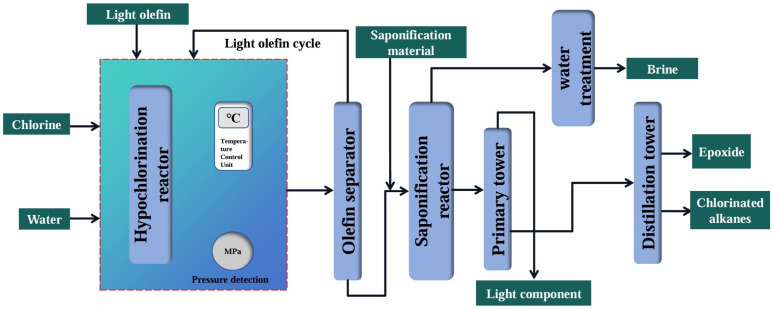
Schematic diagram of the light olefin epoxidation process via the chlorohydrin method.

The chlorohydrin process, which dated back to the 19th century and was the earliest technology for producing epoxides, had a selectivity of over 90% throughout the entire process. The main by-products it generated were chlorinated alkanes produced by the addition reaction and a large amount of brine discharged during the dehydrochlorination process [89]. After that, the process was improved, in which NaOH was used to replace lime milk as the saponification material. This improvement avoided the generation of a large amount of calcium chloride and, thus, led to the formation of a stable epoxide preparation process. As shown in Figure 2, in this process, hypochlorous acid, which was generated by the reaction of the introduced chlorine and water, served as the oxidant. Light olefins were chlorinated to form chlorohydrins in the hypochlorination reactor. After being separated by the olefin separator, they entered the saponification reactor, where the corresponding chlorohydrins reacted with the saponification material to form epoxides. Finally, the purified epoxides were obtained through distillation. This technology was mainly mastered by enterprises, such as China Petroleum and Chemical Corporation, Dow Chemical in the United States, and BASF in Germany. Since it had advantages, such as mature process, simple flow, good selectivity, high safety, low requirement for raw material purity, low investment and cost competitiveness, the chlorohydrin process still accounted for a large proportion in the industrial production of epoxides, and this process was mostly used for the epoxidation of propylene and butylene. However, the chlorohydrin method, which polluted the environment, generated hypochlorous acid, corroded equipment, and consumed a large amount of water and energy, was greatly restricted. In the background of green development in the new era, this process was gradually phased out. Therefore, researchers successively developed various alternative processes to achieve green light olefin epoxidation.

### 2.2. HPPO Method

**Figure 3:**
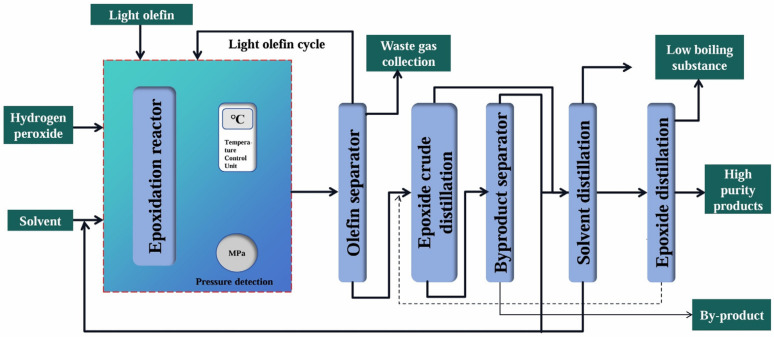
Schematic diagram of the light olefin epoxidation process via the HPPO method.

Among different oxidants, H_2_O_2_ was a safe, inexpensive, easily available and relatively non-toxic oxidant, whose only by-product was water. Light olefins could be epoxidized to prepare epoxides under mild conditions in the presence of H_2_O_2_ [90,91,92,93,94]. The HPPO method, which was pioneered by the Italian company Enichem, was first commercialized by Evonik and SKC. As shown in Figure 3, the introduced H_2_O_2_, which served as the oxidant, was used to carry out the epoxidation of olefins in the epoxidation reactor. After the separation of olefins, the mixture underwent rough distillation and then rectification to obtain the purified epoxides. In this process, both the solvent and olefins could be recycled. Compared with the chlorohydrin method, this process, which had a simple flow, mild reaction conditions, high product yield and relatively few by-products, basically did not produce waste residue and other toxic pollutants, thus belonging to a clean process. The HPPO method, which had developed over the years, had become relatively mature and accounted for a large proportion of the market share. In order to further reduce its production cost, H_2_O_2_ was usually synthesized in situ. Among them, the two main preparation techniques of H_2_O_2_ were the chemical anthraquinone process and the direct synthesis of H_2_ and O_2_ catalyzed by nano-palladium [89].

The HPPO method mostly utilized TS-1 and Schiff–base metal complexes as catalysts. Among them, the HPPO method commercialized by Evonik, which used TS-1 as the catalyst, directly epoxidized propylene with hydrogen peroxide. As a result, the selectivity of the obtained propylene oxide (PO) exceeded 90%, and water was the only by-product. Matias Alvear et al. [95] synthesized ethylene oxide in a laboratory-scale trickle-bed reactor with a commercial TS-1 catalyst, where they used H_2_O_2_ as the oxidant under a wide range of experimental conditions (15–80 °C, 2.5–8.5 bar). They concluded that the catalyst was highly stable and selective during the 150 h operation. The main reaction product was ethylene oxide, whose selectivity generally exceeded 90%. Wenjuan Yan et al. [96] studied the epoxidation of ethylene with H_2_O_2_ as the oxidant over W-KIT-6 and Nb-KIT-6 materials under mild reaction conditions (35 °C, 50 bar). These conditions, which avoided the generation of CO_2_, led to high ethylene oxide (EO) selectivity. The EO yields observed with these materials (30–800 mg_EO_/((g_metal_)·h)), which were of the same order of magnitude as those of the traditional silver (Ag)-based gas-phase ethylene epoxidation method, were remarkable. Xinqing Lu et al. [97] post-synthesized the fluorine-implanted titanosilicate Ti-MWW by fluoridating the conventional Ti-MWW with NH_4_F. This material, whose catalytic performance was significantly enhanced, was used in the liquid-phase epoxidation of ethylene with H_2_O_2_. Baohe Wang et al. [98] introduced a new type of titanosilicate zeolite, HTS-1-X, which had characteristics, such as a high specific surface area, a high titanium content, hierarchical pores, and hydrophobicity. This zeolite, which showed good catalytic ability in the propylene epoxidation reaction, had a high specific surface area that significantly increased the conversion of H_2_O_2_. By controlling the content of phenolic resin, the PO selectivity could reach an optimal value. After being reused for five cycles, the PO selectivity still remained at a relatively high level (88.94%). Yaxian Li et al. [99] designed a nanoscale hierarchical porous zeolite with intracrystalline mesopores. The nano-TS-1 (360 × 190 × 640 nm), which was first synthesized via a seed-assisted method in a tetrapropylammonium bromide (TPABr)-n-butylamine dry-gel system, was post-modified with a pure ammonium fluoride (NH_4_F) solution. When this catalyst was used for propylene epoxidation, the conversion rate of H_2_O_2_ and the selectivity of PO reached 98.4% and 97.6%, respectively. Even after five runs, the catalyst, which maintained stable performance after simple filtration, showed good reusability. Swarup K. Maiti et al. [100] reported a systematic study on the Nb-EISA catalyst and its carbonized version, C-Nb-EISA, which were used for propylene epoxidation under operating conditions similar to those employed in the HPPO method. Compared with other Nb-silicates prepared by the one-pot method and the impregnation method, the Nb-EISA catalyst, which showed excellent activity, stability, and H_2_O_2_ utilization in propylene epoxidation, outperformed Ti-based catalysts significantly in terms of propylene epoxidation. The optimized Nb-EISA catalyst had a PO selectivity and H_2_O_2_ utilization both greater than 99%. Yi Zuo et al. [101] prepared TS-1 using different additives, SBA-15 and silicon carbide. The samples obtained when SBA-15 or silicon carbide were added showed enhanced catalytic activity and stability. Although this process had good prospects, it had a single product and relatively weak risk resistance ability. Hydrogen peroxide, which was highly reactive after being produced in the oxidation unit, led to problems such as transportation safety and storage difficulties. The process flow was long, and the investment cost was relatively high. Therefore, researchers developed a direct epoxidation process with molecular oxygen/air.

### 2.3. Oxygen/Air Direct Oxidation Method

In recent years, molecular oxygen as a terminal oxidant has received increasing attention in the field of epoxidation due to its features, such as abundant reserves, low cost, ready availability and environmental friendliness. In the industrialization of EO production via the direct oxygen oxidation method, the ethylene conversion rate was 7–15% and the EO selectivity was 80–90%. In addition, Benjamin T. Egelske et al. [102] synthesized the Ag/α-Al_2_O_3_ catalyst, which attained 75% selectivity for EO with oxygen as the oxidant. Therefore, given the effectiveness of silver in the epoxidation of ethylene to EO, many studies on the direct preparation of PO took silver as the starting point of research [103]. However, the performance of Ag-based catalysts for PO was not very excellent. For the 14 wt% Ag/CaCO_3_ catalyst, at around 480 K, the turnover frequency of EO formation based on the exposed Ag atoms was in the range of 4–8 × 10^−2^/s, while the TOF of PO was 10-times lower. The corresponding conversion rates of EO and PO were 10% and 15%, and the selectivities were 42% and 9% [6]. Therefore, researchers also developed other metal-based olefin oxidation systems with oxygen. For example, Zhaoning Song et al. [104] designed a three-layer structured S-1/TS-1@dendritic-SiO_2_ as the support for the Au catalyst, achieving excellent catalytic stability (over 100 h), PO selectivity (93.9%) and H_2_ utilization efficiency (26.1%) in the direct epoxidation of propylene with H_2_ and O_2_.

The oxygen/air direct oxidation method was highly regarded by researchers owing to its distinctive features, namely the highest cleanliness, optimal atom utilization rate, abbreviated process flow, and non-generation of by-products. However, aside from the successful industrial production of EO from ethylene via this process, the direct epoxidation of other olefins with oxygen as the oxidant remained at the laboratory stage. This was attributed to several technical bottlenecks impeding its industrial scalability. These included the relatively low conversion and selectivity of PO, as well as the underdeveloped state of catalyst technology. Consequently, with production requirements as the fundamental consideration, researchers devised the olefin epoxidation process, adopting the co-oxidation method.

### 2.4. Co-Oxidation Method

Compared with the direct oxidation method, the co-oxidation method was an olefin epoxidation technology bridged by a co-oxidant. Specifically, the co-oxidant reacted with oxygen to first form an organic peroxide, which, after undergoing separation and purification, reacted with olefins to yield epoxides. The co-oxidation method overcame chlorohydrin method drawbacks like corrosion and high wastewater volume. Additionally, due to market demand for by-products from the co-oxidant, it offered advantages, such as lower costs and reduced environmental impact, making it the epoxidation process with the largest market share and the most epoxidation production lines under construction at that time. According to the differences in co-oxidants, the co-oxidation method is currently mainly divided into the ethylbenzene co-oxidation method, the isobutane co-oxidation method, and the cumene co-oxidation method [105].

#### 2.4.1. Ethylbenzene Co-Oxidation Method

**Figure 4:**
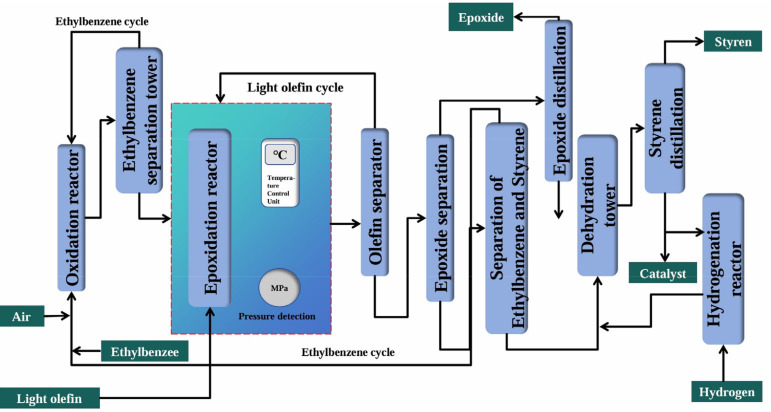
Schematic diagram of the light olefin epoxidation process via the ethylbenzene co-oxidation method.

The ethylbenzene co-oxidation method was an olefin epoxidation approach with molecular oxygen as the oxidant and ethylbenzene as the co-oxidant. Significantly, it co-produced styrene during epoxide synthesis. Styrene, highly demanded in the market, served as a crucial monomer for synthesizing resins, ion-exchange resins, and synthetic rubbers. Additionally, it found application in industries, like pharmaceuticals, dyes, pesticides, and mineral processing [106,107,108]. In 1969, this process was first industrially applied and subsequently put into production as a method for preparing epoxides while co-producing styrene. As depicted in Figure 4, ethylbenzene initially reacted with air in the oxidation reactor to form ethylbenzene hydroperoxide, which, after separation and purification in the ethylbenzene separation tower, served as an oxidant for the olefin epoxidation reaction in the epoxidation reactor. The product then passed through the olefin separator and epoxide separator and was rectified to obtain purified epoxides, with both ethylbenzene and light olefins being recyclable. At that time, ethylbenzene co-oxidation technology was predominantly held by foreign enterprises like Lyondell and Shell, with most domestic production lines established via technology imports. Nevertheless, Wanhua Chemical in China made breakthroughs in four core technologies of this process: ethyl benzyl hydroperoxide (EBHP) preparation, olefin epoxidation, catalytic dehydration, and hydrogenation. By using a rare-earth-modified titanium silicon catalyst, it achieved 94% selectivity for propylene oxide preparation and a 98% yield of styrene.

During the olefin epoxidation process where EBHP served as the oxidant, the catalytic active centers were predominantly high-oxidation-state transition metals of Lewis acids, with the selectivity of epoxides hinging on the Lewis acid and the metal oxidation state [109,110]. R. Martos Calvente et al. [111] prepared a homogeneous complex of Mo (VI) and 4,6-dimethyl-2-mercaptopyrimidine ligand (DMMP) and tested the 1-octene epoxidation performance at 367 K using EBHP as the oxidant to achieve an 86% epoxide selectivity. Laura Barrio et al. [112] prepared molybdenum-containing catalysts by anchoring Mo (VI) groups onto different amino-functionalized silica surfaces and tested the 1-octene epoxidation performance with EBHP as the oxidant, obtaining an 80% selectivity at around 80% EBHP conversion. The ethylbenzene co-oxidation method, capable of co-producing styrene, reduced costs while generating no corrosive substances and little wastewater, thus being relatively more environmentally friendly and overcoming the drawbacks of the chlorohydrin and direct oxidation methods. Nevertheless, it suffered from disadvantages, like a large quantity of required reaction substrates, high operating pressure, and a lengthy process flow.

#### 2.4.2. Isobutane Co-Oxidation Method

**Figure 5:**
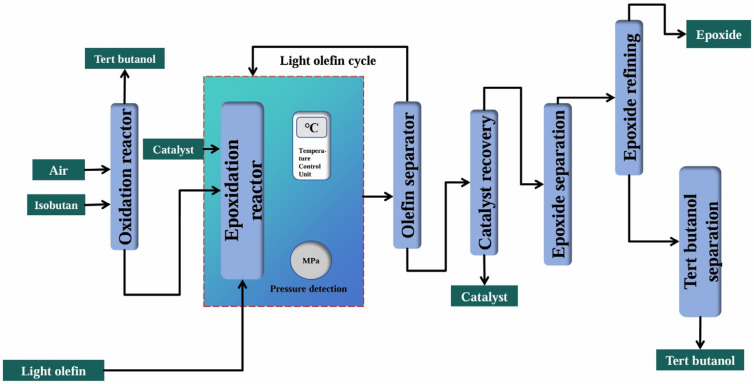
Schematic diagram of the light olefin epoxidation process via the isobutane co-oxidation method.

As shown in Figure 5, the isobutane co-oxidation method, which took isobutane as the raw material, first had the isobutane react with oxygen to generate *tert*-butyl hydroperoxide (TBHP), and then this TBHP reacted with olefins to prepare epoxides while co-producing *tert*-butyl alcohol (TBA). The TBA could be used to prepare methyl *tert*-butyl ether, a high-octane-number fuel additive in vehicle gasoline [113]. Both the isobutane co-oxidation method and the ethylbenzene co-oxidation method were developed by ARCO Chemical during the same period. Currently, the isobutane co-oxidation process technology is mainly in the hands of foreign companies, such as ARCO, Lyondell, and Texaco, with most domestic isobutane co-oxidation technologies being imported from abroad. However, the Shandong Binzhou Group and the Institute of Industrial Research of Tsinghua University achieved the PO/TBA industrialization test in 2020.

In the past, due to its numerous advantages, such as high thermal conductivity, good solubility in polar solvents and neutral pH, the synthesis of TBHP and the epoxidation of olefins using TBHP as an oxidant received increasing attention [114,115,116]. Thomas Willms et al. [117] first studied the oxidation of isobutane in a micro-reactor and compared the product ratios obtained with DTBP and TBHP as initiators. Compared with TBHP at the same concentration, the conversion of DTBP was almost doubled, yet the selectivity of TBHP was higher when using TBHP. With aqueous TBHP, the conversion rate reached up to 6% under supercritical conditions. Industrially, purified TBHP had excellent oxidizing properties and could directly react with olefins in an epoxidation reaction without the addition of a catalyst. However, high-purity TBHP faced issues such as transportation safety, necessitating the introduction of media like water, decane, and nonane to dilute TBHP and the addition of a catalyst to achieve efficient activation of low-concentration TBHP. Zijie Wang et al. [118] prepared a titanium catalyst containing wrinkled mesoporous silica (Ti-WMS) through direct synthesis. With TBHP as an oxidant, they achieved liquid-phase propylene epoxidation with a PO yield and selectivity, reaching 60% and 100%, respectively. In the conditions of 120 °C, with 1,2-dichloroethane as the solvent and a molar ratio of propylene to TBHP of 10:1, the MoO_2_-salen@MCF-S material prepared by Dawei Chen et al. [119] exhibited optimal epoxidation performance. After 1 h, the conversion rate of TBHP was as high as 100%, and the selectivity for PO and TBA reached 94.7% and 84.6%, respectively. The efficient activation of olefins by low-concentration TBHP effectively avoided problems such as transportation and storage during the production process and also prevented the excessive generation of TBA due to the self-decomposition of TBHP during the reaction. However, the isobutane co-oxidation process still accompanied the production of a large number of by-products. The downstream product of *tert*-butyl alcohol, methyl *tert*-butyl ether, was carcinogenic, posing challenges to this process [120,121].

#### 2.4.3. Cumene Co-Oxidation Method

**Figure 6:**
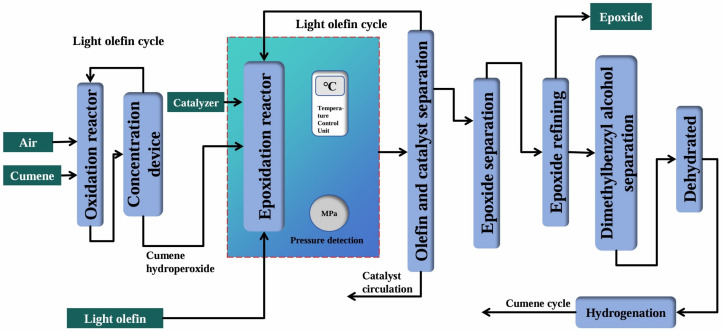
Schematic diagram of the light olefin epoxidation process via the cumene co-oxidation method.

In response to the issue of by-products generated from the ethylbenzene co-oxidation process and the isobutane co-oxidation process, in 2003, Sumitomo Chemical Company of Japan developed an olefin epoxidation method with cumene as a co-oxidant. The cumene oxidation process, with cumene as the raw material, first underwent an air oxidation reaction to obtain cumene hydroperoxide (CHP) and then reacted with olefins to prepare epoxides, as shown in Figure 6. The by-product dimethylbenzyl alcohol produced in the cumene co-oxidation process could be recycled to cumene through hydrogenation, eliminating the generation of by-products in this process. At that time, this technology was mainly in the hands of Sumitomo Chemical. Hongbaoli in China introduced Sumitomo’s technology and started up the propylene epoxidation production line.

Cumene oxidation was typically carried out in the liquid phase. Air or oxygen was used as the oxidant without adding a catalyst, and a small amount of CHP or other substances was added as an initiator to prepare CHP [122]. But the conversion of cumene in this preparation process was relatively low and could not meet industrial demands. Therefore, researchers developed a series of catalysts to achieve the efficient preparation of CHP. Anna Nowacka et al. [123] prepared a Co-Ni bimetallic trimeric acid MOF catalyst via the rapid hydrothermal synthesis method, which was used for the aerobic oxidation of cumene to CHP, and the obtained CHP had a selectivity of 91%. Additionally, Kuo-Tseng Li et al. [124] prepared a Ti/SiO_2_ epoxidation catalyst with a titanium concentration of ≤1.12 μmol/m^2^ by depositing TiCl_4_ on silica gel at 900 °C for 0.5–3 h using the vapor-phase chemical deposition method. With CHP as the oxidant, it was applied to the liquid-phase epoxidation of propylene, and the obtained PO had a selectivity of 97.3%. Zhinan Xia et al. [125] prepared the novel metal-porphyrin framework Mo_2_O_4_(C_48_H_28_N_4_O_8_)·(CH_3_)_2_NH·5H_2_O·2DMF (Mo_2_TCPP) from tetrakis (4-carboxyphenyl) porphyrin (H_4_TCPP) and sodium molybdate dihydrate via the hydrothermal method. With CHP as the oxidant, both the conversion of cyclohexene and the selectivity of cyclohexene oxide were over 99%. The cumene co-oxidation process had broad application prospects due to its advantages, like the absence of by-products and coproducts and the recyclability of the cumene product. However, at that time, the process had not been applied on a large scale, and its process technology was relatively imperfect.

### 2.5. Biomimetic Catalytic Oxidation Method

**Figure 7:**
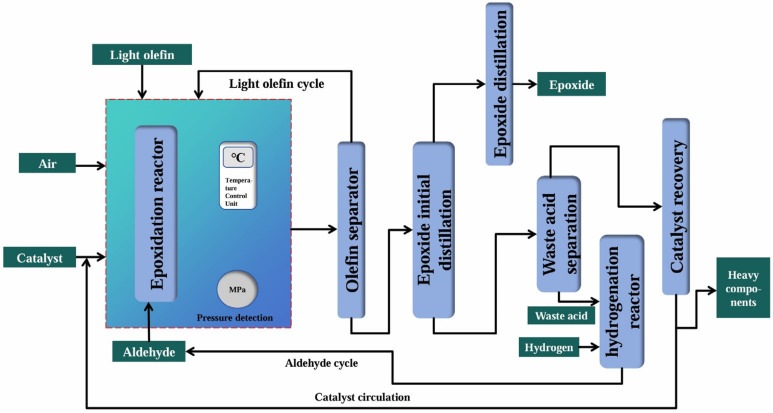
Schematic diagram of the light olefin epoxidation process via the biomimetic catalytic oxidation method.

Organic peroxyacids had strong oxidation activity and exhibited excellent performance towards inert olefins as oxidants [126,127]. Nevertheless, the traditional peroxyacid oxidation method was phased out due to numerous issues, such as the high cost of peroxyacids, strong corrosiveness, and long production processes. Aldehydes, a common reagent in organic chemistry, were inexpensive, readily available, and capable of undergoing auto-oxidation and reacting with molecular oxygen to produce various reactive intermediates [128]. Therefore, researchers conducted studies on biomimetic catalytic epoxidation technology with aldehydes as reducing agents. Specifically, they coupled the peroxyacid oxidation reaction unit, the concentration unit, and the epoxidation unit and developed a biomimetic catalytic oxidation method, integrating aldehydes/olefins/oxygen or air, as shown in Figure 7. This method could activate molecular oxygen and achieve highly efficient epoxidation of olefins under mild conditions. It had advantages, such as a short process and low cost, making it a promising epoxidation technology. In recent years, this technology gradually moved towards industrial application. In addition, Hongbing Ji et al. [129] used manganese porphyrin (MnTPPCl) as a catalyst to achieve the liquid-phase epoxidation of propylene to PO by using benzaldehyde and molecular oxygen. The conversion of propylene and the selectivity of PO could reach 38% and 80%, respectively. Hongbing Ji et al. [130] prepared an olefin epoxidation method with *N*-hydroxyphthalimide (NHPI) as the catalyst, isobutyraldehyde as the reducing agent, and air as the oxidant, and the 40% conversion of 1-butene and the 95% selectivity of BO were realized at ambient temperature. However, it remained to be studied and explored whether the aldehyde-mediated biomimetic catalytic epoxidation process could achieve waste-free acidification throughout the process and co-produce high-value-added chemicals.

In conclusion, through the analysis and discussion of various epoxidation processes in this review, it was evident that each method had its own unique features. Table 1 presents the reaction equations and key characteristics of these methods, and a detailed comparison from this table could guide the selection of appropriate epoxidation techniques in different scenarios, taking into account factors such as efficiency, cost, and environmental impact.

### 2.6. Application of Olefin Epoxidation Processes

#### 2.6.1. Application of Light Olefin Epoxidation Processes

**Figure 8:**
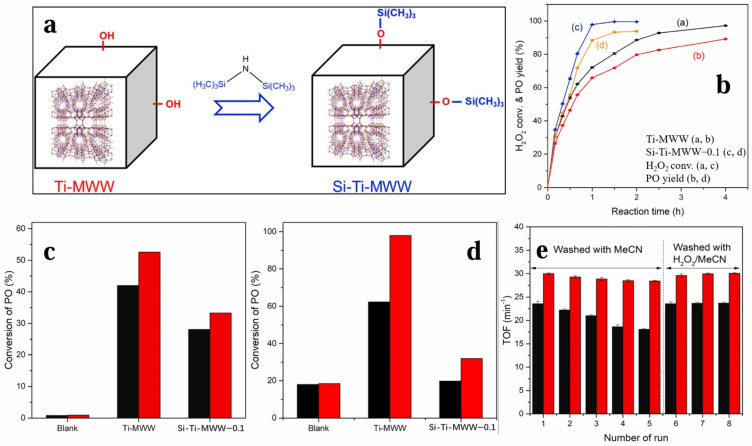
Synthesis of Si-Ti-MWW catalyst and its catalytic performance in propylene epoxidation with H_2_O_2_ as the oxidant, adapted from Tang et al. (2021) [25]: (**a**) flowchart of the trimethylsilylation reaction of Ti-MWW zeolite; (**b**) variation in H_2_O_2_ conversion and PO yield in propylene epoxidation over Ti-MWW and Si-Ti-MWW-0.1 with reaction time; alcoholysis (**c**) and hydrolysis (**d**) of PO with (red) and without (black) H_2_O_2_ over Ti-MWW, Si-Ti-MWW-0.1, and in the absence of any catalyst (blank run), respectively; (**e**) reusability of Ti-MWW (black) and Si-Ti-MWW-0.1 (red) in the propylene epoxidation reaction with H_2_O_2_ as the oxidant.

In the current work, Kang Tang et al. [25] developed trimethylsilylated Ti-MWW zeolite, named Si-Ti-MWW, for the HPPO process. Combining the extensive characterization results of the prepared zeolite, it was found that trimethylsilylation occurred only on the outer surface of the Ti-MWW crystals. This could significantly enhance hydrophobicity without altering the coordination state of titanium species and the structural characteristics of the zeolite. Furthermore, the catalytic performances of Ti-MWW and Si-Ti-MWW catalysts in the propylene epoxidation with H_2_O_2_ as the oxidant were compared. As shown in Figure 8, the results indicated that the solvent decomposition of PO to by-products could be effectively inhibited on the hydrophobic zeolite surface, enabling Si-Ti-MWW to exhibit higher PO selectivity as well as better stability and reusability than the parent Ti-MWW in the HPPO process. Therefore, this research provided a new method for developing highly efficient catalysts for the HPPO process.

**Figure 9:**
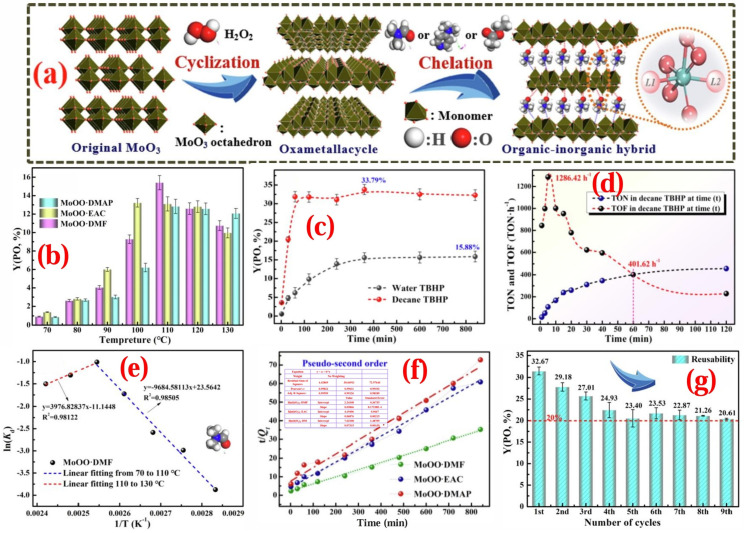
Synthesis of MoOO·DMF catalyst and its catalytic performance in propylene epoxidation with TBHP as the oxidant, adapted from Xiong et al. (2022) [131]: (**a**) synthesis diagram of the catalyst; (**b**) influence of temperature on catalytic performance; (**c**) influence of reaction time of propylene epoxidation over MoOO·DMF by TBHP in the presence of decane and water; (**d**) TON/TOF values at reaction time t of decane-TBHP over MoOO·DMF in a homogeneous system (110 °C); (**e**) the linear fitting (ln(*K*_d_) vs. 1/T) of MoOO·DMF to propylene epoxidation; (**f**) pseudo-second-order models for propylene epoxidation; (**g**) reusability of the catalyst.

Chao Xiong et al. [131] prepared a thermoregulated phase-transfer catalyst (MoOO·DMF) for the liquid-phase propylene epoxidation reaction, with TBHP as the oxidant. MoOO·DMF had the characteristics of both homogeneous and heterogeneous catalysts. It could dissolve in the solvent at higher temperatures and separate from the solvent by lowering the temperature after the reaction. Moreover, MoOO·DMF exhibited excellent epoxidation performance for many olefins, such as light olefins, linear α-olefins, and cyclic olefins. As shown in Figure 9, through the optimization of reaction conditions, the optimal performance of the MoOO·DMF catalyst in the propylene epoxidation reaction was finally obtained. The conversion of propylene was 35.2%, the yield of PO was 33.8%, and the selectivity of PO was 90.6%. When decane-TBHP was used as the oxidant, the TON value of MoOO·DMF increased significantly with an increase in reaction time and reached equilibrium at approximately 1 h. Meanwhile, the TOF increased rapidly in the initial stage, reaching a maximum value (1286.42 h^−1^) at 5 min, and then gradually decreased as the reaction time increased. At the reaction equilibrium, the TOF was still higher than 401 h^−1^. Thermodynamic studies revealed that before 110 °C, the reaction characteristics showed non-spontaneous disorder and an exothermic process. Kinetic studies indicated that the reaction conformed to a pseudo-second-order kinetic model, and the reaction rate was proportional to the square of the concentration of TBHP. MoOO·DMF combined the advantages of homogeneous and heterogeneous catalysts. It showed good stability and reusability in the propylene epoxidation reaction and could be recycled nine times.

#### 2.6.2. Application of Cyclic Olefin Epoxidation Processes

**Figure 10:**
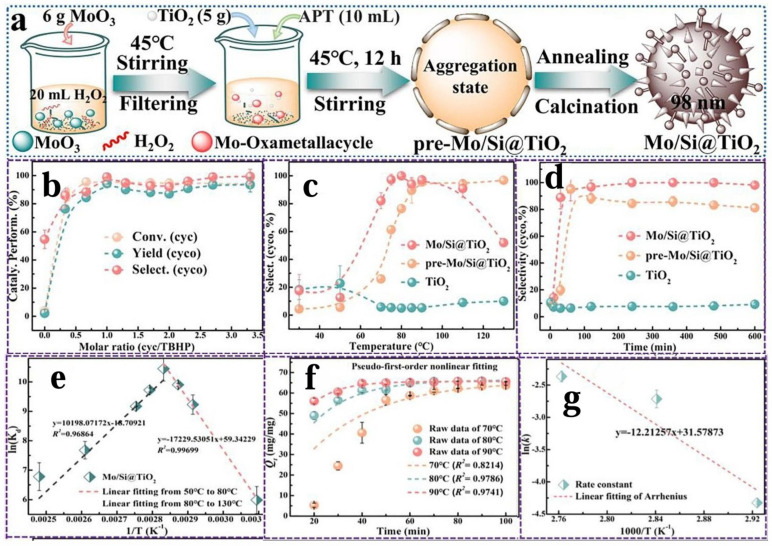
Synthesis of Mo/Si@TiO_2_ catalyst and its catalytic performance in cyclooctene epoxidation with TBHP as the oxidant, adapted from Xiong et al. (2023) [132]: (**a**) diagram of catalyst synthesis; (**b**) molar ratio of TBHP to cyclooctene; (**c**) reaction temperature; (**d**) reaction time; (**e**) the linear fitting (ln(*K*_d_) vs. 1/T) of Mo/Si@TiO_2_ to cyclooctene epoxidation; (**f**) pseudo-first-order models for cyclooctene epoxidation; (**g**) Arrhenius linear fitting.

Chao Xiong et al. [132] synthesized a heterogeneous Mo-based catalyst (Mo/Si@TiO_2_) via a redox strategy. They investigated the physicochemical properties and catalytic behavior of this catalyst using various characterization methods, and they performed the epoxidation reaction using Mo/Si@TiO_2,_ with cyclooctene as the model substrate and TBHP as the oxidant. As shown in Figure 10, through the screening of performance under different conditions, Mo/Si@TiO_2_ exhibited 99% conversion of cyclooctene and 99% selectivity for cyclooctene oxide under the optimal reaction conditions and had good reusability, excellent TBHP consumption efficiency, and wide substrate applicability. Thermodynamics indicated that the cyclooctene epoxidation system catalyzed by Mo/Si@TiO_2_ was endothermic and disordered. The epoxidation rate of cyclooctene on Mo/Si@TiO_2_ followed a pseudo-first-order model, and its apparent activation energy was 101.5 kJ/mol. The study deduced that the *tert*-butylperoxy radicals generated by the activation of TBHP through Mo/Si@TiO_2_ effectively promoted the epoxidation of cyclooctene to its corresponding epoxy product. This work not only provided a reference for the stable loading of Mo-based catalysts but also answered the questions of how to activate TBHP and how to promote olefin epoxidation from the perspective of free radicals.

**Figure 11:**
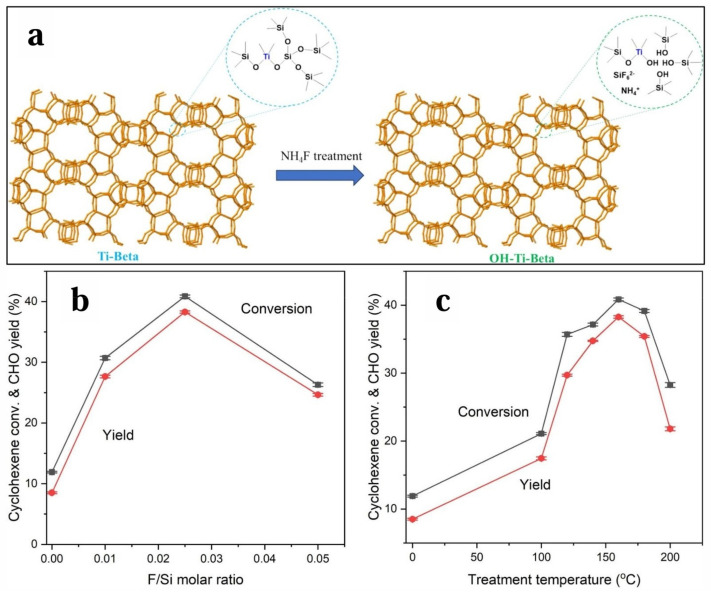
Synthesis of OH-Ti-Beta catalyst and its catalytic performance in cyclohexene epoxidation with H_2_O_2_ as the oxidant, adapted from Xu et al. (2024) [133]: (**a**) flowchart of converting the closed Ti(OSi)_4_ sites to open Ti(OSi)_3_OH sites by treating Ti-Beta with an NH_4_F solution; (**b**) influence of the nominal F/Si molar ratio and (**c**) temperature during the NH_4_F treatment for preparing the OH-Ti-Beta zeolite type on its catalytic performance in the epoxidation of cyclohexene with H_2_O_2_. The symbols for a nominal F/Si molar ratio of 0 and a temperature of 0 °C represent the catalytic results of Ti-Beta.

Bowen Xu et al. [133] successfully synthesized OH-Ti-β zeolite with open Ti(OSi)_3_OH sites by treating Ti-β zeolite with NH_4_F in methanol. During this process, the reaction between the closed Ti(OSi)_4_ sites and NH_4_F generated open Ti(OSi)_3_OH sites, which was confirmed by characterization using various techniques. Benefiting from the stronger Lewis acidity of the open Ti(OSi)_3_OH sites, compared with the parent Ti-Beta, the optimal OH-Ti-Beta zeolite exhibited some superior catalytic performance in the epoxidation of cyclohexene with H_2_O_2_. As shown in Figure 11, during the NH_4_F treatment for preparing the OH-Ti-Beta zeolite, the cyclohexene conversion and cyclohexene oxide yield increased sharply with the increase in the nominal F/Si molar ratio and temperature, reaching their maximum values at a nominal F/Si molar ratio of 0.025 and a temperature of 160 °C, respectively. This work provided some guidance for the development of efficient titanosilicate catalysts for the H_2_O_2_-involved epoxidation of cycloolefins.

#### 2.6.3. Application of Aromatic Olefin Epoxidation Processes

**Figure 12:**
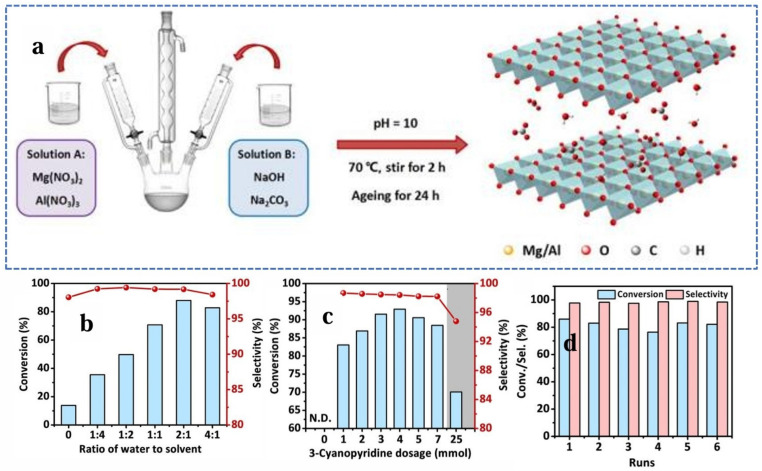
Synthesis of MgAl hydrotalcite catalyst and its catalytic performance in styrene epoxidation with H_2_O_2_ as the oxidant, adapted from Zhang et al. (2023) [134]: (**a**) synthesis diagram of the MgAl hydrotalcite catalyst; optimization of reaction conditions and catalyst stability: (**b**) optimization of the ratio of water to solvent (methanol); (**c**) optimization of the 3-cyanopyridine dosage; (**d**) reusability of the catalyst.

Liwei Zhang et al. [134] reported a tandem-catalyzed highly efficient olefin epoxidation and the intensive production of nicotinamide derivatives. In this reaction, 3-cyanopyridine was proven to be highly efficient and promoted the selective formation of epoxides by with MgAl hydrotalcite as the catalyst. As shown in Figure 12, the epoxidation of styrene was carried out at 50 °C for 3 h with H_2_O_2_ as the oxidant, achieving a selectivity of 98% and a styrene conversion of 93%. Meanwhile, in each reaction cycle, when four equivalents of styrene participated in the reaction, 3-cyanopyridine was simultaneously converted into high-value-added nicotinamide derivatives with a conversion of 87%. Designed experiments demonstrated that the introduction of dehydrogenated peroxyhydroxamic acid, generated from 3-cyanopyridine and a key reactive oxygen species for epoxidation, activated the C=C and promoted the formation of epoxidation products. The generality of the tandem catalytic approach containing base and radical catalysis for this reaction was demonstrated by selective epoxidation of other olefins with similar performance.

**Figure 13:**
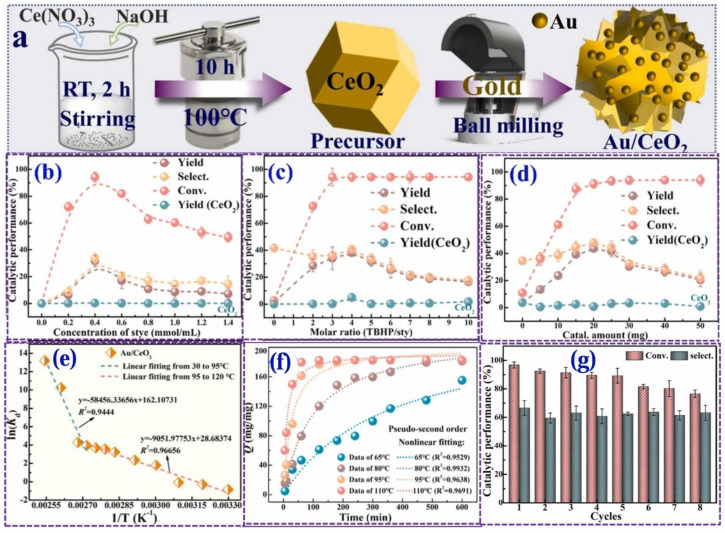
Synthesis of Au/CeO_2_ catalyst and its catalytic performance in styrene epoxidation with TBHP as the oxidant, adapted from Xiong et al. (2023) [135]: (**a**) diagram of catalyst synthesis; (**b**) concentration of styrene; (**c**) molar ratio of TBHP to styrene; (**d**) dosage of the catalyst; (**e**) the linear fitting (ln(*K*_d_) vs. 1/T) of Au/CeO_2_ to styrene epoxidation; (**f**) pseudo-second-order models for styrene epoxidation; (**g**) reusability of the catalyst.

Chao Xiong et al. [135] successfully synthesized ceria-supported coinage metal catalysts (M/CeO_2_, M = Au, Ag, and Cu) via a dry mechanochemical method. In this process, the oxygen vacancies of ceria played a key role in the anchoring of metal nanoparticles. After ball-milling, Au (III) was partially reduced and co-existed on ceria in two valence states (Au^3+^ and Au^0^), enhancing the reactive oxygen species of the prepared catalyst. The catalytic performance of the Au/CeO_2_ catalyst for the styrene epoxidation reaction to prepare styrene oxide (SO) with TBHP as the oxidant was studied. As shown in Figure 13, through the optimization of reaction conditions, the Au/CeO_2_ catalyst exhibited good styrene epoxidation performance with a total styrene conversion of 94% and an SO yield of 63%, and it also had good reusability and substrate scalability. Thermodynamic studies indicated that the styrene epoxidation system catalyzed by Au/CeO_2_ was endothermic and disordered. Catalytic kinetic studies showed that the reaction rate obeyed a pseudo-second-order kinetic equation, and the reaction was dominated by the intrinsic chemical reaction rate on the catalyst surface rather than the mass transfer rate. Its apparent activation energy was 85.46 kJ/mol, which was higher than the generally accepted energy for diffusion-controlled reactions (about 10–15 kJ/mol). Moreover, the Au/CeO_2_ catalyst could maintain good stability and reusability after eight reaction cycles. Based on experimental discussions and a series of characterizations, the mechanism was revealed to be the synergistic catalysis between the reactive oxygen species of the Au/CeO_2_ catalyst and the peroxide radicals generated by the homolytic cleavage of TBHP.

## 3. Overview of Catalysts for Light Olefin Epoxidation

Based on various olefin epoxidation processes, researchers developed a large number of catalysts to achieve efficient epoxidation of light olefins, which were mainly divided into homogeneous catalysts [136,137,138,139,140,141,142,143,144,145] and heterogeneous catalysts [146,147,148,149,150,151,152,153,154,155,156,157,158,159,160,161,162,163,164,165]. This review mainly focuses on processes such as direct oxidation and co-oxidation to give an overview of epoxidation catalysts.

### 3.1. Homogeneous Catalyst

In the homogeneous catalytic system, the catalyst and the reactants could make full contact, making the reaction more efficient and enhancing the product yield and selectivity to a certain extent. The common homogeneous catalysts in light olefin epoxidation mainly included the porphyrin catalyst system [166,167,168,169,170,171,172,173,174,175,176,177] and other metal complex catalysts.

#### 3.1.1. Porphyrin Catalyst System

Porphyrins were an important class of natural macrocyclic organic compounds, and their transition-metal complexes were called metalloporphyrins. Metalloporphyrins were an important class of catalyst for olefin epoxidation, mainly acting through highly reactive metal–oxygen intermediates. The biomimetic catalytic system using metalloporphyrin as a catalyst has been partially applied in the epoxidation of propylene due to its advantages of mild conditions and high efficiency. XianTai Zhou et al. [178] reported a method for the direct catalytic epoxidation of propylene with manganese porphyrin as the catalyst. In the optimal conditions, the conversion of propylene reached 12.7% and the selectivity of PO was 80.5%. Through in situ UV experiments, the high-valent Mn(IV)O porphyrin species was likely the active species of the reaction. XianTai Zhou et al. [179] also reported a method for propylene epoxidation using ruthenium porphyrin as the catalyst and cerium sulfate as the co-catalyst. In optimized conditions, the conversion of propylene was 33.7% with the selectivity of PO being 82.3%. Yang Li et al. [129] reported that, in the presence of benzaldehyde with oxygen as the oxidant, manganese porphyrin was applied to catalytically oxidize propylene to PO in the liquid phase. Manganese (III) porphyrin exhibited excellent activity for the selective oxidation of propylene under mild conditions. The conversion of propylene and the selectivity for PO could reach 38% and 80%, respectively. When the porphyrin catalyst system was used in the olefin epoxidation process, a large amount of reducing agents were involved, leading to the generation of a large quantity of by-products. When aldehyde was used as the reducing agent, a large amount of waste acid was produced concomitantly.

#### 3.1.2. Schiff–Base-Based Metal Complexes

Schiff–base metal complexes exhibited good catalytic activity and could significantly accelerate the reaction rate of olefin epoxidation. Among numerous transition metals, Mo complexes attracted much attention due to their economic viability, environmental friendliness, and commercial feasibility, making Schiff–base Mo complexes stand out in olefin epoxidation reactions. Hamed Mahmoudi et al. [180] prepared a novel ONO Schiff–base dioxomolybdenum(VI) complex for catalyzing the epoxidation of olefins with water as the solvent. They tested the catalytic epoxidation reaction by maintaining a molar ratio of the molybdenum complex, cyclooctene, and TBHP of 1:40:10. This complex showed remarkable effects as a catalyst in the epoxidation of cyclooctene, with a TON exceeding 1400. This catalytic system demonstrated good activity in water and could be used for the epoxidation of various olefins. Mohamed Shaker S. Adam et al. [181] synthesized two cis-bis-dioxomolybdenum oxalylsalicylaldehyde dihydrazone complexes (MoO_2_L_1_ and MoO_2_L_2_) by complexing dioxomolybdenum(VI) acetylacetonate with the bis-Schiff–base chelating ligands oxalylsalicylaldehyde dihydrazone (H_2_L_1_) and p-sodiumsulfonate oxalylsalicylaldehyde dihydrazone (H_2_L_2_), respectively. These complexes showed significant catalytic efficiency in the epoxidation of cyclooctene with H_2_O_2_ or *tert*-butyl hydroperoxide at 85 °C. Under aqueous conditions, MoO_2_L_2_ (with a p-sodiumsulfonate substituent) performed better than MoO_2_L_1_ (without a p-sodiumsulfonate group), highlighting the role of the sodiumsulfonate substituent in the catalytic process of molybdenum chelates.

#### 3.1.3. Other Metal Complex Catalysts

Transition metal-based catalysts showed potential in the selective catalytic conversion of molecules [182]. Among them, Mo complexes had significant advantages, like economy, environmental friendliness, and commercial viability [183]. Their oxidation potential and high Lewis acidity enabled them to promote olefin epoxidation [184,185,186], leading to extensive research on Mo compounds with Schiff–base ligands as efficient homogeneous catalysts [187,188,189]. In addition to Mo, metals such as W, Cr, Re and V could be used in the preparation of metal peroxocyclic complex catalysts [190]. This type of catalyst had excellent highly selective oxygen-transfer ability and was applicable to the epoxidation of nearly all olefins.

### 3.2. Heterogeneous Catalyst

Although homogeneous catalysts could cover the epoxidation of most olefins and achieve high efficiency and selectivity, the complex separation and purification process of homogeneous catalysts, together with the difficulty in recycling and reusing precious metal homogeneous catalysts, greatly increased the production cost. Therefore, researchers turned to the development of heterogeneous catalytic systems, enabling the obtained catalysts to be separated from the reaction solution by simple filtration and reused, thus promoting a reduction in catalyst production costs. The existence of strong metal–carrier interactions made it possible to prepare metal-supported solid catalysts [191]. The heterogeneous catalysts used for the epoxidation of light olefins mainly included supported metal nanocatalysts such as Ag-based, Au-based, and Cu-based [192,193,194,195,196,197,198,199,200,201,202,203,204,205,206,207,208,209,210,211] and other heterogeneous systems.

#### 3.2.1. Supported Metal Nanocatalysts

Nanoparticles became one of the research hotspots in the catalytic field due to their high surface area and ability to provide numerous reaction centers. They could be immobilized on solid supports to control chemical reactions, and the combination of the two ensured high efficiency, high selectivity, high stability, and easy recyclability in both homogeneous and heterogeneous catalysis. The ideal catalytic performance of supported metal nanocatalysts was obtained by microscopically regulating the particle size, surface morphology, metal type, support type, and synergistic effect of support/metal nanoparticles of the catalyst [212]. At that time, supported metal nanoparticles were usually prepared through methods, such as coprecipitation, deposition–precipitation, wet impregnation, and ball milling, and the involved supports mainly included graphene oxide, molecular sieves, polymers, metal–organic frameworks, carbon materials, metal oxides, and non-metallic oxides [213,214,215]. This section introduces the supported metal nanocatalysts applied in the epoxidation of light olefins from the perspective of different metals.

##### Ag-Based Nanocatalysts

**Figure 14:**
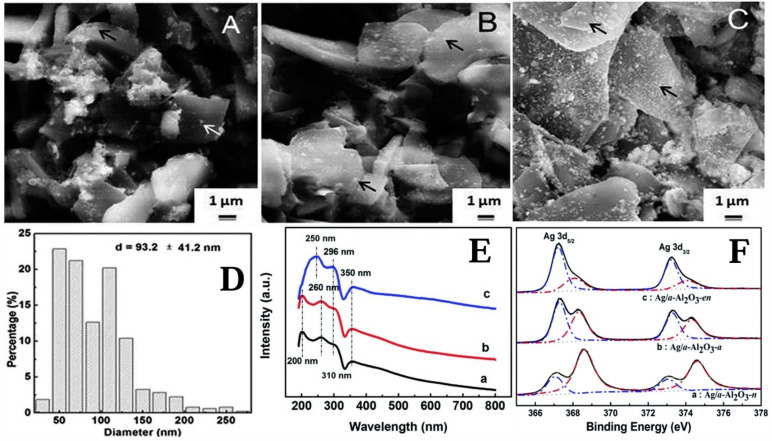
Characterization of Ag/α-Al_2_O_3_ catalysts, licensed under CC-BY [226]: SEM images of (**A**) Ag/α-Al_2_O_3_-n, (**B**) Ag/α-Al_2_O_3_-a, and (**C**) Ag/α-Al_2_O_3_-en catalysts; (**D**) corresponding histogram of Ag particle size distribution on the Ag/α-Al_2_O_3_-en catalyst; (**E**) UV-Vis DRS spectra of a Ag/α-Al_2_O_3_-n, b Ag/α-Al_2_O_3_-a, and c Ag/α-Al_2_O_3_-en; (**F**) XPS spectra of the three catalysts in the Ag 3d region. Arrows in (**A**–**C**) indicate the Ag nanoparticles on α-Al_2_O_3_ support.

Silver-based catalysts were considered the most effective catalysts for the epoxidation of ethylene and propylene [216,217,218]. The epoxidation of ethylene to produce EO was one of the important industrial processes as EO was an essential chemical intermediate that could be further converted into major chemicals, like pharmaceuticals, detergents, plastics and antifreeze [219]. The industrial production of EO was achieved by the gas-phase selective oxidation of ethylene using a supported Ag/Al_2_O_3_ catalyst in a fixed-bed tubular reactor [220,221,222,223]. For the unpromoted silver catalyst, the obtained EO selectivity was only close to 50% [224], and the EO selectivity could exceed 90% after adding promoters such as Cs, Re, Mo inhibitors or CH_2_Cl_2_ [225]. It could be demonstrated that both promoters and inhibitors had a significant impact on the performance of Ag-based catalysts. The addition of inhibitors such as Cs, Re, Mo or promoters like CH_2_Cl_2_ was able to modify the electronic structure and active sites on the catalyst surface. Among them, Cs^+^ interacted with the surface of the Ag/Al_2_O_3_ catalyst, adjusting the electron-cloud distribution of silver atoms and effectively suppressing side reactions. As a result, the selectivity for ethylene oxide was greatly increased from approximately 50% to over 90%. This indicated that promoters played an important role in optimizing the performance of Ag-based catalysts and could enhance the selectivity of the target product by altering the micro-structure of the catalyst. In addition, the selectivity of the Ag/Al_2_O_3_ catalyst for the ethylene epoxidation reaction was largely determined by the geometric structure of Ag nanoparticles, the types of active oxygen species, and the reaction conditions. Xiaolian Jing et al. [226] prepared the Ag/α-Al_2_O_3_ catalyst through a bioreduction method using the camphor tree extract as the reducing agent and AgNO_3_, [Ag(NH_3_)_2_]^+^, and [Ag(en)_2_]^+^ as metal precursors (named n, a, and en, respectively). It can be seen from Figure 14A–D that, compared with Ag/α-Al_2_O_3_-n and Ag/α-Al_2_O_3_-a catalysts, a large number of Ag particles on the Ag/α-Al_2_O_3_-en catalyst are well dispersed on the support, and the size of its Ag particles is 93.2 ± 41.2 nm. According to Figure 14E, the [Ag(en)_2_]^+^ precursor is completely reduced to Ag^0^. The peak of the Ag/α-Al_2_O_3_-en catalyst at the 296 nm band is much larger than those of the other two catalysts, demonstrating the best catalytic activity for ethylene epoxidation. As shown in Figure 14F, the main species on the Ag/α-Al_2_O_3_-en catalyst is Ag^0^. Moreover, the Ag/α-Al_2_O_3_-en catalyst could carry out ethylene epoxidation at 250 °C, obtaining an ethylene conversion of 12% and an EO selectivity of 79.1%.

The silver catalyst successfully epoxidized ethylene into EO, so the silver catalyst became the starting point for research on propylene epoxidation. Shilpi Ghosh et al. [227] applied Ag nanoparticles onto WO_3_ nanorods and performed the epoxidation reaction of propylene with O_2_ as the oxidant. At 250 °C and 2 MPa O_2_ pressure, the propylene conversion was 15.5%, the PO selectivity was 83%, and the yield was 6.1 × 10^−2^ mol/(g_cat_·h). Zuo [228] investigated the modification effect of NaCl, BaCl_2_, LiCl, and NH_4_Cl on the Ag catalyst, and the modification by NaCl and BaCl_2_ had the greatest improvement on the PO yield. The Ag catalyst modified by NaCl (with a feed composition of 90% air and 10% propylene) had a propylene conversion of 18.6% and a PO selectivity of 33.4% at 350 °C. Further increasing the air concentration led to a significant decrease in the PO selectivity (to 10% at 350 °C). Eo Jin Lee et al. [229] studied the modification effect of Mo (0–5 wt%) and W (0–5 wt%) on the 20 Ag/ZrO_2_ catalyst. Catalytic experiments showed that there was a significant correlation between the PO selectivity and the red shift of the Ag 3d_5/2_ binding energy. The optimal catalyst was 20 Ag-3.75 Mo-1.25 W/ZrO_2_, and at 460 °C, the propylene conversion was 12.5% and the PO selectivity was 60%.

##### Au-Based Nanocatalysts

**Figure 15:**
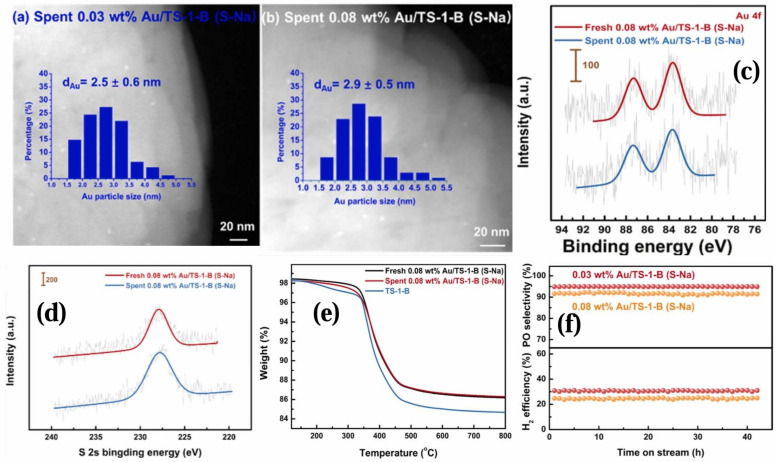
Characterization and stability of Au/TS-1-B (S-Na) catalyst, adapted from Zhang et al. (2022) [238]: HAADF-STEM images and particle size distributions of (**a**) the Au/TS-1-B (S-Na) catalyst and (**b**) the Au/TS-1-B (S-Na) catalyst after reaction; (**c**) Au 4f XPS spectrum; (**d**) S 2s XPS spectrum; (**e**) TGA curve; (**f**) stability.

Haruta et al. [230] and Hutchings et al. [231] independently discovered supported nano-gold heterogeneous catalysts and successfully applied them to many organic conversions. Haruta [232] reported that gold nanoparticles on metal oxides had the ability to chemisorb and dissociate O_2_ below 100 °C. Haruta et al. [233] found that propylene could undergo an epoxidation reaction on the Au/TiO_2_ gas phase with O_2_ and H_2_, and the catalytic behavior of Au/TiO_2_ and its related systems attracted great attention in the industry. There were four important factors in the preparation of highly efficient gold catalysts for the propylene epoxidation reaction. Firstly, it was the preparation method. The deposition–precipitation method could produce selective oxidation [128]. Secondly, it was the support. Among single metal oxides, only Au supported on anatase TiO_2_ selectively produced PO at temperatures below 373 K, while when the Ti-SiO_2_ support was used, Au selectively epoxidized at 473 K with a conversion of about 5% and PO selectivity exceeding 90% [234,235]. Thirdly, it was the size of Au particles. Au particles with a size between 1.4 and 5.0 nm had a relatively high Au^δ-^-Ti^4+^ content, thus obtaining higher propylene conversion and PO selectivity [236]. Finally, it was the additives. Alkaline and alkaline earth salts might play an important role in selectivity and the epoxidation reaction [237]. Zhihua Zhang et al. [238] prepared a highly efficient Au/TS-1-B bifunctional catalyst by simply impregnating a chloride-free sulfur-containing gold precursor (Na_3_Au(S_2_O_3_)_2_) onto uncalcined TS-1. This catalyst showed higher activity than those prepared by impregnating with (sodium) chloride precursors and by the deposition–precipitation with urea (DPU) method. As shown in Figure 15a,b, the diameter of Au particles in the catalyst was nearly the same before and after the reaction, indicating that the Au particles fixed on TS-1-B were very stable during the reaction. According to Figure 15c,d, the intensities of S 2s and Au 4f signals did not show significant decreases before and after the reaction, which indicated that the sulfur substances contained in the Au precursor could serve as protective ligands, thereby promoting the formation of highly dispersed Au substances on uncalcined TS-1. As shown in Figure 15e, the TGA curves of the Au/TS-1-B (S-Na) catalyst were similar before and after the reaction, suggesting that the amount of coke formed during the reaction was very small. In addition, the catalyst exhibited excellent catalytic performance in the catalytic epoxidation of propylene with H_2_ and O_2_. The PO production rate reached 293 g_PO_/(h·g_Au_), and the PO selectivity was approximately 95%, demonstrating excellent gold atom utilization efficiency. In Figure 15f, the Au/TS-1-B (S-Na) catalyst showed good stability for over 42 h. Angeles Pulido et al. [239] studied gold nanoparticles supported on a graphene support. They conducted highly selective epoxidation of propylene using a H_2_/H_2_O/O_2_ mixture and an Au/graphene catalyst. They concluded that the dissociation of O_2_ into two oxygen atoms led to propylene combustion, and this combustion was strongly hindered on this material (Au_1_/G_1V_). The presence of H_2_ promoted the formation of gold hydroperoxide (Au-OOH) species, thus making the production of PO more selective.

##### Cu-Based Nanocatalysts

**Figure 16:**
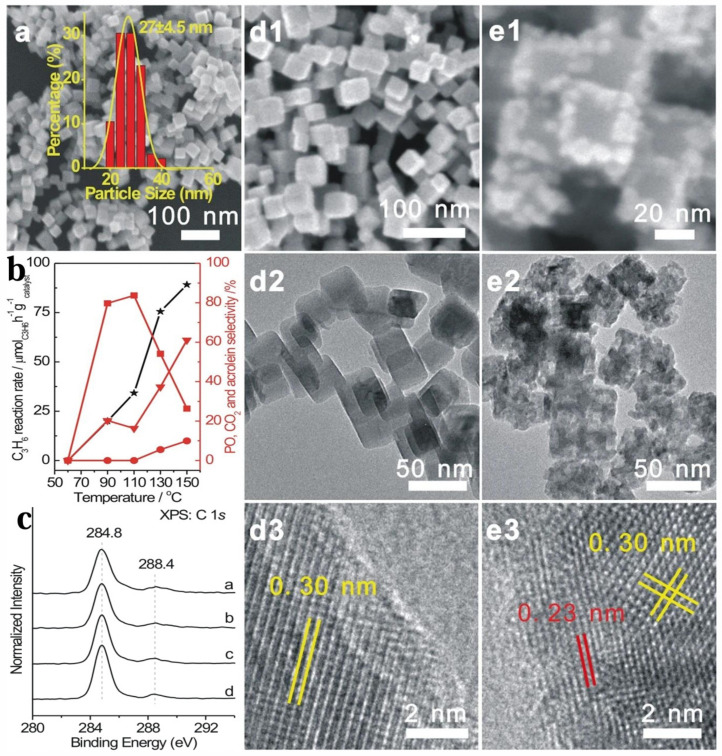
Characterization and performance of c-Cu_2_O-27 catalyst, licensed under CC-BY [12]: (**a**) SEM image and particle size distribution of the c-Cu_2_O-27 catalyst; (**b**) oxidation reaction rates of C_3_H_6_ and O_2_ (black) and selectivities of PO, CO_2_ and acrolein (red) catalyzed by c-Cu_2_O-27; (**c**) C 1s XPS spectra: a c-Cu_2_O-27, b c-Cu_2_O-106, c c-Cu_2_O-774 and d c-Cu_2_O-439; SEM, TEM and HRTEM images of c-Cu_2_O-27 evaluated after C_3_H_6_ oxidation with O_2_ at 90 °C (**d1**–**d3**) and 150 °C (**e1**–**e3**).

Supported Cu-based catalysts showed potential in the catalytic epoxidation of propylene to produce propylene oxide. The feasibility of epoxidizing propylene with SiO_2_-supported Cu-based catalysts was preliminarily determined. The obtained PO selectivity was 32.6% and the propylene conversion rate was 0.3%, and it was proved that the active phase of the reaction was Cu^0^ [240]. But, research by Monnier and Hartley pointed out that Cu^0^ was a poor epoxidation catalyst because under oxidative reaction conditions, it was easily oxidized to Cu_2_O or CuO, and both of these phases were either inactive or non-selective [241]. Hua et al. [242] studied the crystal plane-controlled selectivity of Cu_2_O during the propylene oxidation process and concluded that the one-coordinated Cu on Cu_2_O (111), three-coordinated O on Cu_2_O(110), and two-coordinated O on Cu_2_O(100) were regarded as the catalytically active sites for the production of acrolein, PO, and CO_2_, respectively. The PO selectivity on Cu_2_O (110) was up to 20% at most, which made the crystal face engineering of Cu_2_O an effective strategy for developing PO selective catalysts. Long et al. [243] found that by adding 5 wt% CuO_x_ and 2 wt% RuO_x_ onto SiO_2_, an efficient propylene epoxidation catalyst could be obtained. At a C_3_H_6_ conversion rate of 5.3%, the PO selectivity was 36%. They speculated the substance leading to PO formation was the complex formed by the direct contact of CuO_x_ and RuO_x_ nanoparticles. Seubsai et al. [244] combined CuO with RuO_2_ and modified it with NaCl, TeO_2_, Cs_2_O, and TiO_2_. The catalyst showed the best performance when the Ru/Cu ratio was 3 and it was modified with NaCl. However, the catalyst deactivated rapidly because the vapor formed during the reaction led to the loss of Cl^−^. Jianyi Lei et al. [245] found through research that the perovskite (LaCoO_3_) modified by sodium chloride and loaded with CuO was active in the direct oxygen epoxidation reaction of propylene, and with the assistance of citric acid, they further prepared LaCo_x_Cu_1−x_O_3−_ modified by alkali metal salts to enhance its catalytic performance. Among them, LaCo_0_._8_Cu_0_._2_O_3−δ_ exhibited the best catalytic performance. At a relatively low temperature (250 °C), the PO production rate was 60.4 g_PO_/(kg_cat_·h) (with a PO selectivity of 10.2% and a propylene conversion of 12.0%). Wei Xiong et al. [12] successfully explored 27 nm fine cubic Cu_2_O nanocrystals with high-density active sites at the Cu_2_O edges as highly selective catalysts for the propylene epoxidation reaction. According to Figure 16(a,d1–d3,e1–e3), the morphology of the c-Cu_2_O-27 catalyst before and after the reaction could be seen. The synthesized uniform surfactant-free cubic Cu_2_O nanocrystals had a size of 27 ± 4.5 nm and were labeled as c-Cu_2_O-27. The BET specific surface area of c-Cu_2_O-27 was 25.5 m^2^/g. The lattice fringes of 0.30 and 0.23 nm in Figure 16(d3,e3) correspond to the interplanar spacings of the Cu_2_O {110} (JCPDS card number 78-2076) and CuO {111} (JCPDS card number 89-5899) crystal planes, respectively. The XPS spectrum (Figure 16c) showed that only amorphous carbon and carbonate existed on the surfaces of various c-Cu_2_O catalysts. PO was selectively produced at temperatures between 90 and 110 °C, with a selectivity of over 80%. In addition, in the CuO_x_/SiO_2_ system, Cs^+^, as an alkali-metal ion additive, formed a strong interaction with CuO_x_ nanoparticles [246]. This interaction eliminated the Lewis acidity of CuO_x_ nanoparticles and inhibited the side reactions, where propylene oxide isomerized to allyl alcohol and was then oxidized to acrolein. As a result, the selectivity for propylene oxide was significantly improved. This demonstrated the important regulatory role of promoters in copper-based catalysts on reaction selectivity, optimizing the reaction pathway by influencing the surface properties of the catalysts.

##### Other Supported Metal Nanocatalysts

In addition to the aforementioned Ag-based, Au-based, and Cu-based nanocatalysts, there were also supported metal nanocatalysts, such as Mo, Ti, W, and Nb, applied in the epoxidation reaction of light olefins. Song et al. [247] used silica-supported molybdenum oxide as a catalyst for the propylene epoxidation reaction with oxygen in a reactor system composed of the volumes of a catalytic bed and a post-catalytic bed. In the condition of MoO_x_/SiO_2_ with a SiO_2_ concentration of 0.255 mmol/g, at 5 atm and 573 K, the propylene conversion rate reached as high as 18% and the PO selectivity was 44%. Chao Xiong et al. [131] prepared a temperature-controlled phase-transfer catalyst (MoOO·DMF) for liquid-phase propylene epoxidation with TBHP as an oxidant. The catalyst had a selectivity of 90.6% for PO (catalyst/propylene = 0.7 mol‰) and a yield of 1286.42 h^−1^. The Tatsumi group [248] described the Ti-MCM-41 catalyst that could form PO when loaded with nitrates. They proposed the possible role of nitrates in activating molecular oxygen to form epoxides. Murata et al. [249] also synthesized MCM-41 catalysts containing Ti and Al and found that Ti-containing catalysts were more effective, possibly due to the synergistic relationship between Ti species and acid sites. Yan et al. [250] observed that mesoporous silicate materials doped with Nb, namely Nb-KIT-5, Nb-MCM-48, and Nb-TUD-1, exhibited remarkable ethylene epoxidation activity under mild operating conditions (35 °C, 50 bar) with H_2_O_2_ as the oxidant and methanol as the solvent, and under these conditions, no carbon dioxide was detected as a by-product.

#### 3.2.2. Other Heterogeneous Systems

In addition, the researchers also developed heterogeneous catalyst systems represented by phase-transfer catalysts, single-atom catalysts, and nanocluster catalysts and achieved relatively good catalytic performance. Phase-transfer catalysts could transfer reactants from one phase to another in a multiphase reaction system, thus promoting the progress of the reaction. This mechanism of action greatly improved the reaction efficiency and overcame the problem of difficult mass transfer between reactant phases in traditional multiphase reactions. Igor Tyablikov et al. [213] reported that the epoxidation of propylene with hydrogen peroxide was catalyzed using 2,2,2-trifluoroethyl benzoate immobilized on SBA-15 mesoporous silica as the catalyst. The obtained propylene selectivity was 100%, achieving a PO yield of 10%. Mirza Cokoja et al. [27] used hydrophobic imidazolium-based ionic liquid (IL) as a catalyst and H_2_O_2_ as an oxidant to epoxidize light olefins in water. Among them, imidazolium perrhenate could both transfer hydrophobic substrates to the aqueous phase and act as the actual catalyst. After the reaction ended, in the absence of H_2_O_2_, the IL catalyst formed a third phase in addition to the lipophilic products and water and could be easily recycled.

Single-atom catalysts demonstrated excellent performance in olefin epoxidation reactions due to their unique atomic-level dispersion characteristics. Hongqiang Jin et al. [251] synthesized various densely loaded M-SACs (M = Fe, Co, Ni, Cu, Zn, Ru, and Ir) with a loading amount of up to 35.5 wt% through a general and reliable strategy. Taking the epoxidation of trans-stilbene with O_2_ as a model reaction, it was found that the reaction rate and mass-specific activity of the 21.2 wt% highly loaded Co SAC were 10-times and 30-times those of the 5.4 wt% Co SAC, respectively. A series of experiments and theoretical studies revealed that in high-density Co SACs, the electronic structure of cobalt atoms was altered through charge redistribution, resulting in a decrease in Bader charge and an increase in the d-band center. These changes were proven to be more favorable for the activation of O_2_ and trans-stilbene. Qingshan Zhao et al. [252] presented a novel core–shell confinement strategy for preparing single-atom iron catalysts (Fe-N/S-C) on carbon nanoshells for olefin epoxidation reactions. By encapsulating polydopamine-coated nano-Fe_2_O_3_ in sulfur-rich petroleum asphalt and undergoing one-step heat treatment, an atomically dispersed Fe-N_3_S_1_ structure with asymmetric coordination was carefully designed. This catalyst achieved a conversion rate of 80.2% and a selectivity as high as 93.6% in the styrene epoxidation reaction. Experimental results and theoretical calculations showed that the negative charge induced by sulfur doping effectively regulated the electronic distribution of the active center, enhanced the adsorption/desorption and activation of reaction substrates, and, thus, accelerated the catalytic process in the selective oxidation reaction.

Nanostructured materials had a high specific surface area. They could provide more active sites, accelerate the reaction process, and have excellent stability. They could still maintain good catalytic performance under harsh reaction conditions, thus prolonging the service life of the catalyst. Ali Dadashgholi Niatouri et al. [253] successfully synthesized various transition-metal-substituted Keggin-type polyoxometalate/Ni-Al layered double-hydroxide/graphene oxide (PWM/Ni-Al LDH/GO; M = Fe, Ni, Cu, Mn, Co, Cr, Zn) nanocomposites and applied them in olefin epoxidation. Among them, the PWFe/Ni-Al LDH/GO nanocomposite showed the best catalytic activity in the epoxidation reactions of various olefins in the presence of hydrogen peroxide. This catalyst could also be reused at least five times in the epoxidation reaction of styrene without a significant loss of catalytic activity. Jingbo Yuan et al. [254] synthesized a core–shell resin structure with a rich structure and excellent thermal stability through a polymerization reaction. By immobilizing imidazolium ionic liquids with different carbon-chain lengths on the surface and using HCO exchange, they prepared metal-free and halogen-free core–shell catalysts. In the epoxidation reaction system, the catalyst exhibited significant conversion and selectivity and also demonstrated strong recyclability in heterogeneous reactions. According to the analysis of kinetic parameters, the reaction orders of styrene, TBHP, and the catalyst in the epoxidation reaction were all approximately 1. The reference factor of this reaction was calculated to be 6.7 × 10^9^ (L^2^·mol^−2^·min^−1^), and the activation energy was 48.9 kJ/mol, obtained through the analysis of the reaction rates at different temperatures.

In this section, we explored catalysts for the epoxidation of light olefins, covering two major categories: homogeneous catalysts and heterogeneous catalysts. Although homogeneous catalysts had high activity and selectivity, there were problems of difficult separation and recovery. This not only increases the production cost but might also lead to the waste of catalysts and environmental pollution. New research directions could explore the development of homogeneous catalysts with self-separation properties or adopt immobilization techniques to load homogeneous catalysts onto solid carriers, thus achieving their separation from the reaction system and enabling repeated use. Heterogeneous catalysts had advantages in terms of separation and repeated use, but there were issues, such as metal agglomeration, loss of active sites, and relatively low reaction activity. In response to these problems, the highly dispersed state of the metal active components could be achieved by precisely controlling the preparation process of the catalysts, thereby improving the stability and activity of the catalysts. It was also possible to enhance the interaction between the metal and the carrier and reduce the occurrence of metal agglomeration by optimizing the carrier materials and performing surface modification. In addition, during the research process of olefin epoxidation, the reaction mechanism should be analyzed in more detail, and efficient catalysts should be designed for the required individual reactions based on the general mechanism.

## 4. Reaction Mechanism of Light Olefin Epoxidation

### 4.1. Ethylene Epoxidation Mechanism with Silver as the Catalyst (L-H Mechanism and E-R Mechanism)

**Figure 17:**
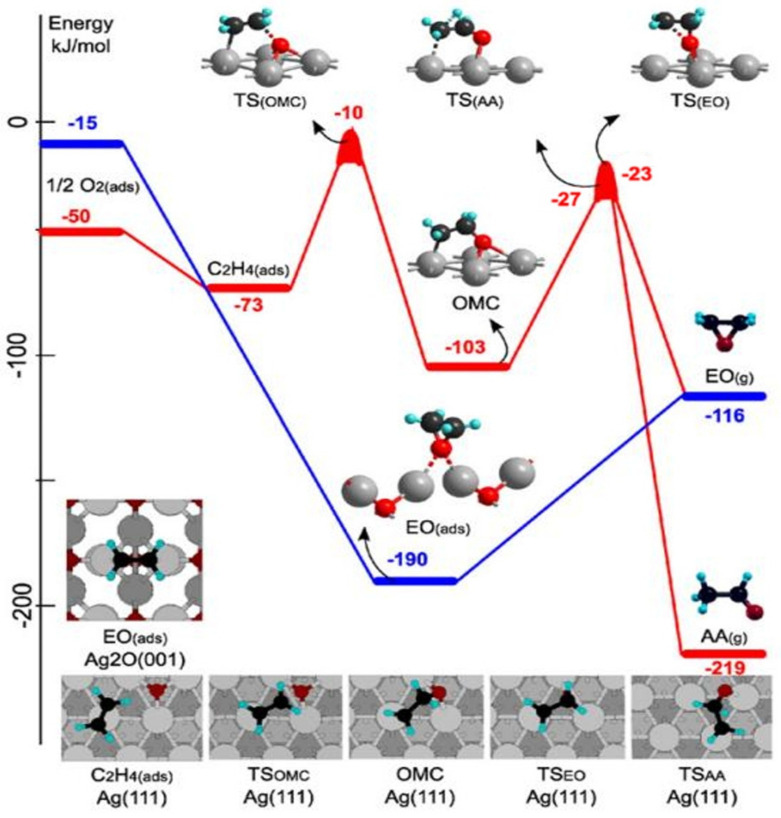
Epoxidation mechanism of ethylene with silver as the catalyst: Reaction energy diagram: Paths of EO and AA formation via the OMC intermediate on the Ag (111) surface (red line (**upper part**)) and the path of direct EO formation on the Ag_2_O (001) surface (blue line (**lower part**)). The figure below represents the top view of reaction intermediates. Energies are with respect to the surface + 1/2 O_2_ (g) + C_2_H_4_ (g)). Color code: Ag gray; O red (dark gray); C black, H cyan (light gray), adapted from Ozbek et al. (2011) [261].

At that time, the common catalytic ethylene oxidation mechanism was the oxametallacycle (OMC) pathway [255,256,257,258,259]. Ethylene and adsorbed oxygen reacted to form a surface OMC intermediate through a fully surface-mediated Langmuir–Hinshelwood (L-H) mechanism (left) or an Eley–Rideal (E-R) mechanism (right). Subsequently, EO and acetaldehyde (AA) were produced through the parallel isomerization reaction between OMC and AA. In reaction conditions, the silver catalyst might not have been pure metal and instead had a thin silver oxide layer. Furthermore, the selectivity of EO directly depended on the surface oxygen coverage, and the OMC pathway on the metallic Ag surface could not even address this issue [260]. Van Santen et al. [261,262,263] investigated the mechanism of ethylene epoxidation on the silver oxide surface, which essentially represented the limit of high surface and subsurface oxygen concentrations. As shown in Figure 17 their DFT calculations indicated that in the absence of oxygen vacancies, the direct oxidation of ethylene was possible, thus obviating the need for a surface OMC intermediate. The bridging oxygen (Ag-O-Ag) was identified as the electrophilic oxygen capable of attacking the C-C bond to directly form EO. In this reaction system, the oxygen coverage on the surface of the silver catalyst was a key factor affecting the selectivity of EO. When the surface oxygen coverage was appropriate, it was conducive to the formation of EO through the OMC pathway. However, when oxygen vacancies were present, the reaction tended to produce AA, resulting in a decrease in the selectivity of the ethylene oxidation reaction. This was because the presence of oxygen vacancies changed the electronic structure of the catalyst surface, causing the activation energy barriers of the reaction to change. The activation energy barrier for the formation of AA was lower than that for the formation of EO, thus influencing the reaction selectivity. In addition, the geometric structure of silver nanoparticles also affected their adsorption capacities for ethylene and oxygen, thereby impacting the reaction activity. Smaller silver particles usually had a higher specific surface area, which could provide more active sites and was beneficial for the progress of the reaction. However, it might also lead to changes in selectivity, which need to be comprehensively considered in practical applications.

### 4.2. 2-Methylpropene Epoxidation Mechanism over Mo(O_2_)_2_@RT with TBHP as Oxidant (Free Radical Mechanism)

**Figure 18:**
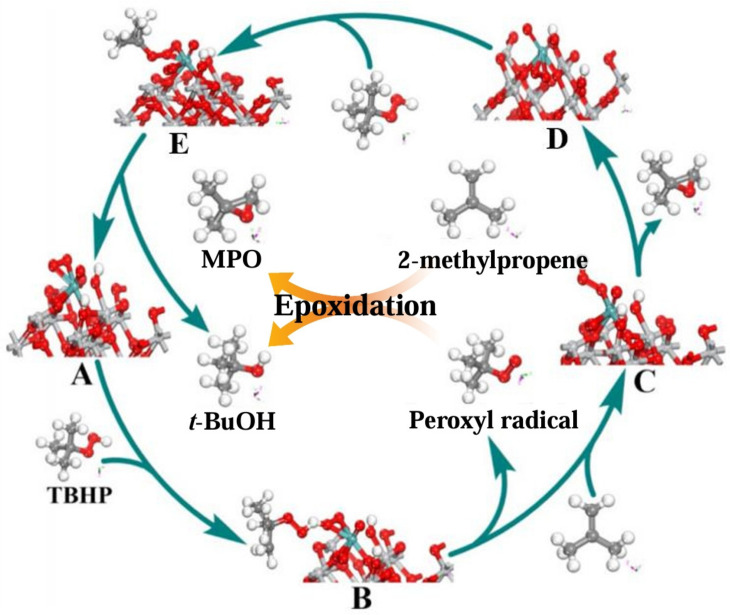
Epoxidation mechanism of 2-methylpropene with Mo(O_2_)_2_@RT as the catalyst and TBHP as the oxidant: (A) Mo(O_2_)_2_@RT, (B) an intermediate in the interaction between TBHP and Mo(O₂)₂@RT, (C) Mo-O-O-H species, (D) Mo=O species, (E) the intermediate state formed after the Mo=O species acquires reactive oxygen species, adapted from Xiong et al. (2023) [264].

In this study, a Mo-based catalyst (Mo(O_2_)_2_@RT) was prepared by transferring the reactive oxygen of H_2_O_2_ to molybdenum oxide through an oxidative pretreatment strategy and combining it with rutile. Its epoxidation performance for 2-methylpropene with TBHP as the oxidant was then evaluated [264]. The catalyst exhibited excellent catalytic activity in the epoxidation of 2-methylpropene to 1,2-epoxy-2-methylpropane (MPO; selectivity: 99.7%; yield: 92%). The mechanism of 2-methylpropene epoxidation on Mo(O_2_)_2_@RT and TBHP was studied. TBHP was activated by Mo(O_2_)_2_@RT to generate highly active *tert-*butyl peroxides radicals, and these radicals achieved the epoxidation of 2-methylpropene to produce MPO. As shown in Figure 18, first, the O-O bond in Mo(O_2_)_2_@RT (A) was attacked by TBHP and then broken, generating a *tert-*butyl peroxide radical and a Mo-O-O-H species (C). Subsequently, the Mo-O-O-H species reacted with 2-methylpropene to form MPO, and the Mo-O-O-H species was transformed into a Mo=O species (D) and an H radical. At the same time, the *tert-*butyl peroxide radical directly participated in the epoxidation of 2-methylpropene, producing MPO and a *tert*-butyl oxygen radical, which then reacted with the H radical to form *tert*-butanol. Finally, in the oxidation of TBHP, the Mo=O species obtained reactive oxygen species from TBHP, reforming the molybdenum peroxide ring species (A) and, thus, entering the next round of epoxidation.

In this reaction system, the surface properties of the Mo(O_2_)_2_@RT catalyst had a crucial impact on the reaction selectivity and acti.vity. The surface structure of the catalyst was an important factor determining its activation ability towards TBHP. The catalyst with specific surface properties could guide the *tert*-butyl oxygen radicals and Mo-O-O-H species to preferentially undergo the epoxidation reaction with 2-methylpropene, thus effectively suppressing the occurrence of other side reactions and ensuring a high selectivity for MPO. In addition, the appropriate distribution of active sites and the electronic-cloud structure could enhance the interaction between Mo(O_2_)_2_@RT and TBHP, effectively promoting the cleavage of the O-O bond in TBHP. As a result, more *tert*-butyl oxygen radicals were generated, significantly enhancing the reaction activity. However, if the active sites were too dense, it would strengthen the interaction between the intermediate species, potentially triggering unnecessary side reactions and having an adverse effect on the reaction selectivity.

### 4.3. Propylene Epoxidation Mechanism over CuO_x_/SiO_2_ with O_2_ as Oxidant (High-Valence Metal Oxo Mechanism)

**Figure 19:**
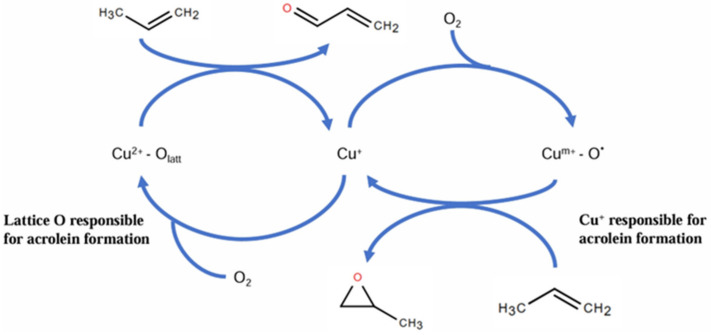
Schematic diagram of the epoxidation mechanism of the Cs-modified CuO_x_/SiO_2_ system, adapted from He et al. (2013) [246].

Cs^+^ was found to be one of the most effective alkali-metal ion additives in the CuO_x_/SiO_2_ system [246]. The Lewis acidity of CuO_x_ particles led to the isomerization of PO to allyl alcohol, which was then oxidized to acrolein. However, Cs^+^ formed a stronger interaction with CuO_x_ nanoparticles, eliminating this acidity and suppressing the isomerization of PO. A schematic diagram of the mechanism of propylene epoxidation in the Cs^+^ modified CuO_x_/SiO_2_ catalyst is shown in Figure 19. Cu^2+^ combined with lattice oxygen (O_latt_) and then reacted with propylene to form acrolein while generating Cu^+^. The Cu^+^ sites activated molecular oxygen to form reactive oxygen, denoted as Cu^m+^-O^*^ in the figure, which was responsible for the epoxidation of propylene to PO.

In this reaction system, the addition of Cs^+^ played a crucial role in changing the surface properties of the CuO_x_/SiO_2_ catalyst. Moreover, the concentration and activity of oxygen species on the catalyst surface were closely related to the surface properties. The catalyst with suitable surface properties could stabilize the active oxygen species (Cu^m+^-O^*^), ensuring that it maintained high activity during the reaction with propylene and effectively promoting the formation of PO. In addition, the distribution and particle size of CuO_x_ particles on the surface of the CuO_x_/SiO_2_ catalyst were important factors affecting the reaction activity. Generally, smaller CuO_x_ particles had a larger specific surface area, which could provide more active sites for the reaction. This was beneficial for the adsorption of propylene and oxygen on the catalyst surface, thereby promoting the progress of the reaction and increasing the reaction activity. However, when the particle size of CuO_x_ particles was too small, it would lead to a decrease in their stability. During the reaction process, agglomeration or oxidation was likely to occur. These changes would reduce the number of effective active sites and instead have a negative impact on the reaction activity.

### 4.4. Propylene Epoxidation Mechanism over MoOO·DMF with TBHP as Oxidant (Free Radical and Peroxide Mechanisms)

**Figure 20:**
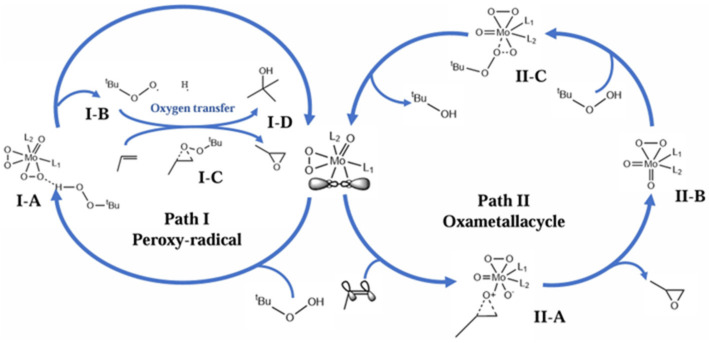
Mechanism of propylene epoxidation to PO with TBHP as the oxidant over MoOO·DMF, adapted from Xiong et al. (2022) [131].

Free radical reactions were considered to be one of the important mechanisms for olefin epoxidation reactions. With TBHP as the oxidant and MoOO·DMF as the catalyst for the epoxidation of propylene, the mechanism was the selective oxygen transfer from the *tert-*butyl peroxide radical and the Mo-O-O bridge in MoOO·DMF to propylene. The Mo-O-O bridge of the catalyst was the key active center for the activation of the C=C bond in propylene and the O-H bond in TBHP, thereby enhancing the selective transfer of oxygen in the propylene epoxidation reaction [131]. As shown in Figure 20, in the radical pathway (from I-A to I-D), TBHP was converted into *tert-*butyl peroxides radicals under the catalysis of MoOO·DMF. These radicals then epoxidized propylene to generate PO and *tert*-butanol. In the OMC catalytic pathway, the cleavage of the Mo-O-O bridge facilitated the transfer of oxygen to propylene, producing PO and the II-B intermediate. Additionally, the II-B intermediate could be further recycled to its initial state through the oxygen transfer from TBHP (II-C).

Among them, the active sites on the surface of the MoOO·DMF catalyst had an appropriate electron-cloud density and spatial structure, which could interact more effectively with TBHP, promote the cleavage of the O-O bond, generate more *tert-*butyl peroxide radicals, and enhance the reaction activity. In addition, specific groups on the surface of the MoOO·DMF catalyst could form weak interactions with propylene molecules, making the adsorption of propylene on the surface selective. As a result, propylene preferentially reacted with reactive oxygen species, reducing the occurrence of side reactions.

### 4.5. Butene Epoxidation Mechanism over TS-1 with H_2_O_2_ as Oxidant (L-H Mechanism)

**Figure 21:**
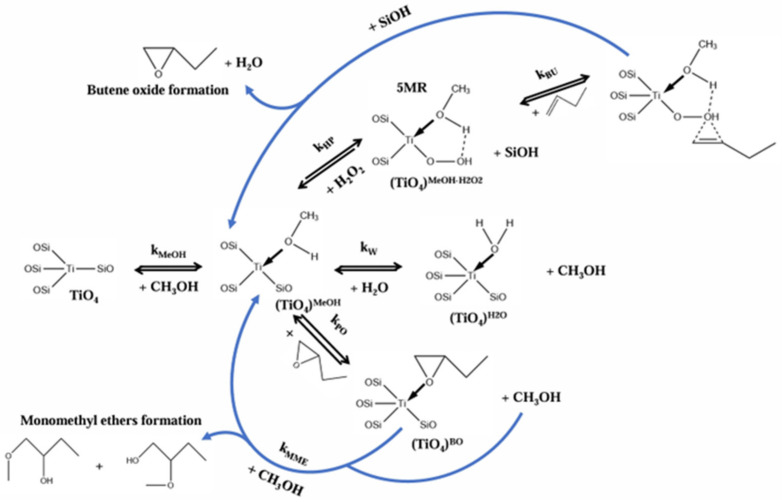
Reaction mechanisms proposed for the adsorption of different substances on tetrahedrally coordinated titanium, adapted from Zuo et al. (2023) [14].

The then widely accepted mechanism of olefin epoxidation with TS-1 and H_2_O_2_ was based on unimolecular adsorption. In this mechanism, H_2_O_2_ was first adsorbed on the “TiO_4_” sites, and then the olefin in the liquid phase was adsorbed on the oxygen atom of H_2_O_2_ to produce an epoxide. Yi Zuo et al. [14] coated TS-1 on specially designed stainless-steel supports and prepared a monolithic TS-1 catalyst. This catalyst was used in the epoxidation reaction of 1-butene, namely, a strongly exothermic reaction that produced butene oxide in a fixed-bed reactor. The reaction mechanism is shown in Figure 21. In the formation process of butene oxide, the starting materials TiO_2_ and CH_3_OH, with the participation of H_2_O and H_2_O_2_, underwent a series of reactions to form (TiO_4_)^MeOH·H^_2_^O^_2_. Subsequently, an intermediate product containing Ti(OH)_2_O_2_ was generated, and, finally, butene oxide was obtained. During the formation process of monomethyl, OH reacted with CH_3_OH, passing through the intermediate product (TiO_4_)^BO^, and finally formed monomethyl. Different arrows are used in the figure to indicate the reaction directions and conditions, and the reaction rate constants are labeled. The oxygen anions in methanol, water, BO, and H_2_O_2_ occupied the vacant orbitals in “TiO_4_”, forming (TiO_4_)^MeOH^, (TiO_4_)^H^_2_^O^, (TiO_4_)^BO^, and (TiO_4_)^MeOH·H^_2_^O^_2_. Among them, the five-membered ring structure (5MR)(TiO_4_)^MeOH·H^_2_^O^_2_ was the active center of the epoxidation reaction.

In this reaction, the crystal structure of TS-1 and the distribution of TiO_4_ sites on its surface determined its adsorption capacity for H_2_O_2_ and butene. The density and activity of TiO_4_ sites directly influenced the adsorption amount and activation degree of H_2_O_2_. If the density of TiO_4_ sites was high and their activity was appropriate, more H_2_O_2_ could be adsorbed and activated more easily, thus enhancing the reaction activity. In addition, groups such as silanol groups on the surface of TS-1 could form specific interactions with butene and H_2_O_2_, making the adsorption and reaction of reactants on the surface selective. This allowed the epoxidation reaction to occur preferentially rather than other competitive reactions, ensuring a high selectivity for butene oxide.

## 5. Progress and Prospects of Green Chemistry in the Epoxidation of Olefins

Against the backdrop of the global heightened concern for environmental protection and sustainable development, the concept of green chemistry has penetrated more deeply into the field of olefin epoxidation reactions. Researchers dedicated themselves to developing more environmentally friendly and efficient olefin epoxidation technologies, and a series of advancements have been achieved. These advancements were mainly focused on cutting-edge technology areas, such as biobased catalysts, electrochemical epoxidation, and photocatalytic epoxidation. Meanwhile, a substantial amount of research work was carried out, and certain results were obtained in aspects, such as enhancing catalyst performance (e.g., improving stability and selectivity), realizing the effective recycling of catalysts, minimizing waste generation, and increasing energy efficiency.

Biobased catalysts, as one of the important research directions in green chemistry, received extensive attention for their unique catalytic mechanisms and environmentally friendly characteristics. Biobased catalysts represented by enzymes can achieve the epoxidation of olefins with high selectivity under mild reaction conditions, significantly reducing the occurrence of side reactions and meeting the strict requirements of green chemistry for atom economy and environmental friendliness. Jia Jing Li et al. [265] reported a method for the chemoselective and enantiodivergent epoxidation of unactivated olefins using an engineered P450DA monooxygenase. P450DA variants, obtained through X-ray structure-guided directed evolution based on P450DA-WT and P450DA-M3, switched the reactivity from the native allylic C-H bond hydroxylation to the epoxidation of C=C bonds. They exhibited excellent chemoselectivity (up to 99% epoxidation selectivity) and enantioselectivity (up to >99:1 er), thus generating a variety of versatile and enantiomerically enriched epoxides. However, biobased catalysts still faced problems, such as poor stability, high production costs, and difficulties in large-scale preparation at that time. These factors limited their widespread application in industrial production to a certain extent.

Electrochemical epoxidation technology, as another important research hotspot, achieved the epoxidation of olefins by precisely regulating the electrochemical reaction conditions. This technology has numerous advantages. For example, it could reduce the dependence on traditional chemical oxidants and lower the emission of harmful chemicals. When powered by renewable energy sources, it could significantly improve energy efficiency and achieve sustainable energy supply. Moreover, the reaction usually occurred under mild conditions, effectively reducing energy consumption and safety risks. Hao Wu et al. [266] disclosed a non-halide-mediated electrochemical epoxidation of olefins, in which water was used as the oxygen source to form epoxides. This method, catalyzed by (TMP)MnCl (TMP = tetramesitylporphyrin), exhibited excellent efficiency and could achieve the epoxidation of aromatic and aliphatic olefins with excellent Faradaic efficiency (up to 89%). Suyeon S. Kim et al. [267] proposed an alternative electrocatalytic epoxidation reaction, using [CoIII(TAML)]^−^ (TAML = tetraamido macrocyclic ligand) as the molecular catalyst. This catalyst could use water as the oxygen atom source and efficiently and selectively epoxidize a variety of olefins. Among them, [CoIII(TAML)]^−^ achieved a Faradaic efficiency of over 60% and a selectivity of over 90% for the epoxidation of cyclohexene. However, it should not be overlooked that this technology faced challenges such as high electrode material costs and relatively low reaction rates, which urgently needed to be addressed through interdisciplinary research in materials science, electrochemical engineering, and other fields. Therefore, developing low-cost and high-performance electrode materials and increasing the reaction rate were the key research directions for electrochemical epoxidation technology at that time for its future development.

Photocatalytic epoxidation technology has made remarkable progress in recent years. This technology utilizes the active species generated by photocatalysts under light irradiation to drive the epoxidation reaction of olefins. The photocatalytic epoxidation reaction uses light as the energy source, has mild reaction conditions, and can use oxygen in the air as the oxidant, avoiding the environmental pollution problems caused by traditional oxidants. Yiming Huang et al. [49] prepared copper nanoparticles (CuNPs) supported on titanium nitride (TiN) without adding other stabilizers. These nanoparticles were not only stable in the air but also selectively catalyzed the epoxidation of various olefins under light irradiation using oxygen or even air as a mild oxidant. Sebastiano Gadolini et al. [268] explored an innovative photocatalytic method, which anchored an iron-salt-type complex (FeSalenCl_2_) onto pristine graphitic carbon nitride (C_3_N_4_). The resulting catalyst [C_3_N_4−FeCl_(Salen)]_Chem_ showed promising catalytic performance in the selective epoxidation of cyclic and linear olefins using oxygen as the oxidant. However, the activity and selectivity of photocatalysts need further improvement. Problems such as low light efficiency and the susceptibility of photocatalysts to photo corrosion limit the large-scale application of this technology. Thus, in-depth research is still required to overcome these issues. Therefore, developing efficient and stable photocatalysts and improving the utilization efficiency of light were the keys to promoting the development of photocatalytic epoxidation technology.

In the research field of green chemistry for olefin epoxidation reactions, the optimization of catalyst performance, waste minimization, and energy efficiency improvement were always the core points. By precisely regulating the microstructure of catalysts, such as using advanced material preparation techniques to control the crystal structure, surface morphology, and distribution of active sites, the stability and selectivity of catalysts could be effectively improved. The generation of waste was reduced by improving the reaction process and optimizing the catalysts. For example, the HPPO method used hydrogen peroxide as the oxidant and basically produced no toxic pollutants, such as waste residues. However, in actual production, there were still problems of unreacted raw materials and intermediate product residues. To further minimize waste, it was necessary to optimize the reaction conditions (such as adjusting the reactant ratio, temperature, time, etc.) and develop efficient separation technologies to reduce the residues. The improvement in energy efficiency was also crucial. In the existing epoxidation processes, the energy input could be reduced, and the energy utilization efficiency could be improved by optimizing the reaction conditions and developing new catalysts. For instance, the biomimetic catalytic oxidation method carried out the reaction under mild conditions and had obvious advantages in energy consumption compared with traditional processes. However, the catalytic efficiency of the biomimetic catalytic oxidation method in large-scale production needed to be improved. The energy efficiency could be further enhanced by optimizing the structure of biomimetic catalysts and exploring new reaction paths and processes to promote the process of green olefin epoxidation reactions.

In conclusion, green chemistry has made a lot of progress in the field of olefin epoxidation, but it still faces challenges. Future research needs to focus on overcoming these challenges, further optimizing existing technologies, and developing new green epoxidation processes to promote the development of olefin epoxidation reactions towards a greener and more efficient direction, providing strong support for the sustainable development of the chemical industry.

## 6. Conclusions

This review summarizes the research progress in the preparation of epoxides via light olefin epoxidation in recent years. It elaborates in detail on three aspects: reaction processes, catalysts, and reaction mechanisms. Due to high costs, strong corrosiveness, and environmental concerns, the traditional chlorohydrin process has gradually been phased out under the background of green development in the new era. Although the HPPO process features high selectivity and efficiency, issues, such as high investment costs, high transportation safety risks, difficult storage and transportation, and complex process flow, limit its widespread application. Therefore, aiming at the goals of green, safe, and sustainable development, researchers have successively developed various new green olefin epoxidation processes. However, some of the new processes are still in the laboratory or small-scale trial stage, facing challenges such as immature technology and lack of large-scale application. Future research needs to further deepen the understanding of the reaction mechanism and design more efficient catalysts to promote the industrialization process of these green processes. Looking ahead, with the increasing demand for environmentally friendly technologies and efficient resource utilization, research in the field of light olefin epoxidation will continue to focus on developing cleaner and more economical production processes, providing strong technical support for achieving the sustainable development goals of the chemical industry.

## Figures and Tables

**Table 1:** Comparison of different epoxidation processes.

Epoxidation Processes	Items	Details
Chlorohydrin Method	Reaction equation	$R-\mathrm{CH}={\mathrm{CH}}_{2}+{\mathrm{Cl}}_{2}+H_{2}O\overset{\mathrm{catalyst}}{\to }R-\mathrm{CH}\left(\mathrm{OH}\right)-{\mathrm{CH}}_{2}\mathrm{Cl}+\mathrm{HCl}$ $R-\mathrm{CH}\left(\mathrm{OH}\right)-{\mathrm{CH}}_{2}\mathrm{Cl}+\mathrm{NaOH}\overset{\mathrm{catalyst}}{\to }R-{\mathrm{CHCH}}_{2}O+\mathrm{NaCl}+H_{2}O$
	Advantages	The process was mature, the flow was simple, the selectivity was high, and the requirement for raw material purity was low.
	Disadvantages	The process consumed a large amount of water, generated a great deal of wastewater and waste residue, polluted the environment, corroded the equipment, and had high energy consumption.
HPPO Method	Reaction equation	$R-\mathrm{CH}={\mathrm{CH}}_{2}+H_{2}O_{2}\overset{\mathrm{catalyst}}{\to }R-{\mathrm{CHCH}}_{2}O+H_{2}O$
	Advantages	The process flow was simple, the product yield was high, few by-products were generated, and basically no pollutants such as waste residue were generated
	Disadvantages	The product was single, the risk-resistance ability was weak, there were transportation safety problems and storage difficulties, the process flow was long, and the investment cost was high.
Oxygen/Air Direct Oxidation Method	Reaction equation	$2R-\mathrm{CH}={\mathrm{CH}}_{2}+O_{2}\overset{\mathrm{catalyst}}{\to }2R-{\mathrm{CHCH}}_{2}O$
	Advantages	The process had a high atomic utilization rate, a short process flow, no by-products, a wide range of raw material sources and relatively low costs.
	Disadvantages	The process had relatively harsh reaction conditions, under—developed catalysts and technical bottlenecks in industrial promotion.
Ethylbenzene Co-oxidation Method	Reaction equation	$C_{6}H_{5}{\mathrm{CH}}_{2}{\mathrm{CH}}_{3}+O_{2}\overset{\mathrm{catalyst}}{\to }C_{6}H_{5}\mathrm{CH}\left(\mathrm{OOH}\right){\mathrm{CH}}_{3}$ $R-\mathrm{CH}={\mathrm{CH}}_{2}+C_{6}H_{5}\mathrm{CH}\left(\mathrm{OOH}\right){\mathrm{CH}}_{3}\overset{\mathrm{catalyst}}{\to }R-{\mathrm{CHCH}}_{2}O+C_{6}H_{5}\mathrm{CH}\left(\mathrm{OH}\right){\mathrm{CH}}_{3}$
	Advantages	The process had little environmental pollution, and the co-products like styrene could share the cost.
	Disadvantages	The technological process was long, the pressure was high, and the reaction conditions were harsh.
Isobutane Co-oxidation Method	Reaction equation	${\mathrm{CH}}_{3}\mathrm{CH}{\left({\mathrm{CH}}_{3}\right)}_{2}+O_{2}\overset{\mathrm{catalyst}}{\to }{\left({\mathrm{CH}}_{3}\right)}_{3}\mathrm{COOH}$ $R-\mathrm{CH}={\mathrm{CH}}_{2}+{\left({\mathrm{CH}}_{3}\right)}_{3}\mathrm{COOH}\overset{\mathrm{catalyst}}{\to }R-{\mathrm{CHCH}}_{2}O+{\left({\mathrm{CH}}_{3}\right)}_{3}\mathrm{COH}$
	Advantages	The process had little environmental pollution, and the co-products like *tert*-butanol could share the cost.
	Disadvantages	The technological process was long, the pressure was high, and the reaction conditions were harsh.
Cumene Co-oxidation Method	Reaction equation	$C_{6}H_{5}\mathrm{CH}{\left({\mathrm{CH}}_{3}\right)}_{2}+O_{2}\overset{\mathrm{catalyst}}{\to }C_{6}H_{5}C{\left({\mathrm{CH}}_{3}\right)}_{2}\mathrm{OOH}$ $R-\mathrm{CH}={\mathrm{CH}}_{2}+C_{6}H_{5}C{\left({\mathrm{CH}}_{3}\right)}_{2}\mathrm{OOH}\overset{\mathrm{catalyst}}{\to }R-{\mathrm{CHCH}}_{2}O+C_{6}H_{5}C{\left({\mathrm{CH}}_{3}\right)}_{2}\mathrm{OH}$
	Advantages	There was less wastewater, less corrosion of the equipment, and there were no by-products (cumene could be recycled).
	Disadvantages	The technological process was complex, and the investment cost was high.
Biomimetic Catalytic Oxidation Method	Reaction equation	$2\mathrm{RCHO}+O_{2}\overset{\mathrm{catalyst}}{\to }2\mathrm{RCOOOH}$ $R-\mathrm{CH}={\mathrm{CH}}_{2}+\mathrm{RCOOOH}\overset{\mathrm{catalyst}}{\to }R-{\mathrm{CHCH}}_{2}O+\mathrm{RCOOH}$
	Advantages	The reaction conditions were mild, the selectivity was high, and it was relatively environmentally friendly.
	Disadvantages	The cost was relatively high, and the catalytic efficiency in large-scale industrial production might be insufficient to meet the requirements, with the presence of a scale-up effect.

## Data Availability

Data are contained within the article.

## References

[1] Amini M. (2016). Efficient and selective oxidation of olefins and alcohols using nanoparticles of WO_3_-supported manganese oxides (W_1−x_Mn_x_O_3_). Korean J. Chem. Eng..

[2] Hu C., Zhang L., Zhang J., Cheng L., Zhai Z., Chen J., Hou W. (2014). Template-free method to prepare porous Cu-containing nanotubes with a good catalytic performance for styrene epoxidation. Appl. Surf. Sci..

[3] Amini M., Haghdoost M.M., Bagherzadeh M. (2014). Monomeric and dimeric oxido–peroxido tungsten(VI) complexes in catalytic and stoichiometric epoxidation. Coord. Chem. Rev..

[4] Yan W., Zhang G., Yan H., Liu Y., Chen X., Feng X., Jin X., Yang C. (2018). Liquid-Phase Epoxidation of Light Olefins over W and Nb Nanocatalysts. ACS Sustain. Chem. Eng..

[5] Huš M., Grilc M., Teržan J., Gyergyek S., Likozar B., Hellman A. (2023). Going Beyond Silver in Ethylene Epoxidation with First-Principles Catalyst Screening. Angew. Chem. Int. Ed..

[6] Khatib S.J., Oyama S.T. (2015). Direct Oxidation of Propylene to Propylene Oxide with Molecular Oxygen: A Review. Catal. Rev..

[7] Li T., Zuo Y., Guo Y., Yang H., Liu M., Guo X. (2020). Highly stable TS-1 extrudates for 1-butene epoxidation through improving the heat conductivity. Catal. Sci. Technol..

[8] Pu T., Tian H., Ford M.E., Rangarajan S., Wachs I.E. (2019). Overview of Selective Oxidation of Ethylene to Ethylene Oxide by Ag Catalysts. ACS Catal..

[9] Pinaeva L.G., Noskov A.S. (2020). Prospects for the Development of Ethylene Oxide Production Catalysts and Processes (Review). Pet. Chem..

[10] Iyer K.R., Bhan A. (2023). Particle size dependence of ethylene epoxidation rates on Ag/α-Al_2_O_3_ catalysts: Why particle size distributions matter. J. Catal..

[11] Yu B., Ayvalı T., Raine E., Li T., Li M.M.-J., Zheng J., Wu S., Bagabas A.A., Tsang S.C.E. (2019). Enhanced propylene oxide selectivity for gas phase direct propylene epoxidation by lattice expansion of silver atoms on nickel nanoparticles. Appl. Catal. B Environ..

[12] Xiong W., Gu X.-K., Zhang Z., Chai P., Zang Y., Yu Z., Li D., Zhang H., Liu Z., Huang W. (2021). Fine cubic Cu_2_O nanocrystals as highly selective catalyst for propylene epoxidation with molecular oxygen. Nat. Commun..

[13] Arvay J.W., Hong W., Li C., Delgass W.N., Ribeiro F.H., Harris J.W. (2022). Kinetics of Propylene Epoxidation over Extracrystalline Gold Active Sites on AU/TS-1 Catalysts. ACS Catal..

[14] Zuo Y., Zhang B., Li T., Zhang M., Fan J., Liu M., Xu J., Yang C., Yang H., Guo X. (2023). Highly stable monolithic titanium silicalite-1 catalyst for 1-butene epoxidation. AIChE J..

[15] Liu Y., Zhao C., Sun B., Zhu H., Xu W. (2021). Preparation and modification of Au/TS-1 catalyst in the direct epoxidation of propylene with H_2_ and O_2_. Appl. Catal. A Gen..

[16] Horstmann S., Gardeler H., Fischer K., Köster F., Gmehling J. (2001). Vapor Pressure, Vapor−Liquid Equilibrium, and Excess Enthalpy Data for Compounds and Binary Subsystems of the Chlorohydrin Process for Propylene Oxide Production. J. Chem. Eng. Data.

[17] Myszkowski J., Milchert E., Bartkowiak M., Pełech R. (2010). Utilization of waste chloroorganic compounds. Pol. J. Chem. Technol..

[18] Myszkowski J., Milchert E., Paździoch W., Pełech R. (2007). Formation of environmentally friendly chloroorganic compounds technology by sewage and by-products utilization. Pol. J. Chem. Technol..

[19] Liu D., Wang R., Yu Y., Chen Z., Fang N., Liu Y., He M. (2023). Chemical deactivation of titanosilicate catalysts caused by propylene oxide in the HPPO process. Catal. Sci. Technol..

[20] Blanco-Brieva G., de Frutos-Escrig M.P., Martín H., Campos-Martin J.M., Fierro J.L.G. (2012). Selective hydrogenation of hydrogen peroxide in the epoxidation effluent of the HPPO process. Catal. Commun..

[21] Liu X., Liu J., Xia Y., Yin D., Steven R.K., Mao L. (2019). Catalytic performance of TS-1 in oxidative cleavage of 1-alkenes with H_2_O_2_. Catal. Commun..

[22] Russo V., Tesser R., Santacesaria E., Di Serio M. (2013). Chemical and Technical Aspects of Propene Oxide Production via Hydrogen Peroxide (HPPO Process). Ind. Eng. Chem. Res..

[23] Zeb A., Park C., Son M., Baek A., Cho Y., Kim D., Rampogu S., Lee G., Kwak Y.-S., Park S.J. (2020). Integration of virtual screening and computational simulation identifies photodynamic therapeutics against human Protoporphyrinogen Oxidase IX (HPPO). Arab. J. Chem..

[24] Triandafillidi I., Kokotou M.G., Lotter D., Sparr C., Kokotos C.G. (2021). Aldehyde-catalyzed epoxidation of unactivated alkenes with aqueous hydrogen peroxide. Chem. Sci..

[25] Tang K., Hou W., Wang X., Xu W., Lu X., Ma R., Fu Y., Zhu W. (2021). Enhanced catalytic performance of trimethylsilylated Ti-MWW zeolites for the liquid-phase epoxidation of propylene with H_2_O_2_. Microporous Mesoporous Mater..

[26] Sulimov A.V., Ovcharova A.V., Ovcharov A.A., Flid V.R., Leont’eva S.V., Bruk L.G., Pastukhova Z.Y., Flid M.R. (2016). Study of alkene epoxidation in the presence of extruded silicalite titanium. Russ. Chem. Bull..

[27] Cokoja M., Reich R.M., Wilhelm M.E., Kaposi M., Schäffer J., Morris D.S., Münchmeyer C.J., Anthofer M.H., Markovits I.I.E., Kühn F.E. (2016). Olefin Epoxidation in Aqueous Phase Using Ionic-Liquid Catalysts. ChemSusChem.

[28] Engelmann X., Malik D.D., Corona T., Warm K., Farquhar E.R., Swart M., Nam W., Ray K. (2019). Trapping of a Highly Reactive Oxoiron(IV) Complex in the Catalytic Epoxidation of Olefins by Hydrogen Peroxide. Angew. Chem. Int. Ed..

[29] Li L., Song H.-J., Meng X.-G., Yang R.-Q., Zhang N. (2018). Efficient epoxidation reaction of terminal olefins with hydrogen peroxide catalyzed by an iron (II) complex. Tetrahedron Lett..

[30] Majetich G., Hicks R., Sun G.-r., McGill P. (1998). Carbodiimide-Promoted Olefin Epoxidation with Aqueous Hydrogen Peroxide. J. Org. Chem..

[31] Li P., Gao J., Shi J., Wang H., Xing X., Ren J., Meng Y., Wang L., Lv B. (2022). Insights into the effect of oxygen vacancies on the epoxidation of 1-hexene with hydrogen peroxide over WO_3−x_/SBA-15. Catal. Sci. Technol..

[32] Rezaeifard A., Jafarpour M., Haddad R., Feizpour F. (2017). {Mo72Cr30} nanocluster as a novel self-separating catalyst for hydrogen peroxide olefin epoxidation. Catal. Commun..

[33] Borrell M., Costas M. (2018). Greening Oxidation Catalysis: Iron Catalyzed Alkene syn-Dihydroxylation with Aqueous Hydrogen Peroxide in Green Solvents. ACS Sustain. Chem. Eng..

[34] Ai C., Zhu F., Wang Y., Yan Z., Lin S. (2019). SO_2_F_2_-Mediated Epoxidation of Olefins with Hydrogen Peroxide. J. Org. Chem..

[35] Wang T., Jing X., Chen C., Yu L. (2017). Organoselenium-Catalyzed Oxidative C-C Bond Cleavage: A Relatively Green Oxidation of Alkenes into Carbonyl Compounds with Hydrogen Peroxide. J. Org. Chem..

[36] Chen Z., Luck R.L. (2016). Oxidation of olefins using atmospheric oxygen atoms initiated by tert-butylhydroperoxide or hydrogen peroxide with silver nanoparticles deposited on MCM-41 as catalysts. Green Chem..

[37] Mao S., Budweg S., Spannenberg A., Wen X., Yang Y., Li Y.-W., Junge K., Beller M. (2022). Iron-Catalyzed Epoxidation of Linear α-Olefins with Hydrogen Peroxide. ChemCatChem.

[38] Hong M., Min J., Wang S. (2018). Metal-Free Epoxidation of Internal and Terminal Alkenes with tert-Butyl Hydroperoxide/Isobutyraldehyde/Oxygen System. ChemistrySelect.

[39] Bose I., Zhao Y. (2022). Site-Selective Catalytic Epoxidation of Alkenes with Tunable Atomic Precision by Molecularly Imprinted Artificial Epoxidases. ACS Catal..

[40] Vanoye L., Wang J., Pablos M., de Bellefon C., Favre-Réguillon A. (2016). Epoxidation using molecular oxygen in flow: Facts and questions on the mechanism of the Mukaiyama epoxidation. Catal. Sci. Technol..

[41] Koya S., Nishioka Y., Mizoguchi H., Uchida T., Katsuki T. (2012). Asymmetric Epoxidation of Conjugated Olefins with Dioxygen. Angew. Chem. Int. Ed..

[42] Iwahama T., Hatta G., Sakaguchi S., Ishii Y. (2000). Epoxidation of alkenes using alkyl hydroperoxides generated in situ by catalytic autoxidation of hydrocarbons with dioxygen. Chem. Commun..

[43] Yamada T., Takai T., Rhode O., Mukaiyama T. (1991). Direct Epoxidation of Olefins Catalyzed by Nickel(II) Complexes with Molecular Oxygen and Aldehydes. Bull. Chem. Soc. Jpn..

[44] Abbasi V., Hosseini-Monfared H., Hosseini S.M. (2017). A heterogenized chiral imino indanol complex of manganese as an efficient catalyst for aerobic epoxidation of olefins. New J. Chem..

[45] Wang Q., Wang L., Mi Z. (2005). Influence of Pt-Pd/TS-1 Catalyst Preparation on Epoxidation of Olefins with Hydrogen Peroxide. Catal. Lett..

[46] Luz I., León A., Boronat M., Llabrés i Xamena F.X., Corma A. (2013). Selective aerobic oxidation of activated alkanes with MOFs and their use for epoxidation of olefins with oxygen in a tandem reaction. Catal. Sci. Technol..

[47] Zheng Y., Shen Q., Li Z., Jing X., Duan C. (2022). Two Copper-Containing Polyoxometalate-Based Metal-Organic Complexes as Heterogeneous Catalysts for the C-H Bond Oxidation of Benzylic Compounds and Olefin Epoxidation. Inorg. Chem..

[48] Tebandeke E., Coman C., Guillois K., Canning G., Ataman E., Knudsen J., Wallenberg L.R., Ssekaalo H., Schnadt J., Wendt O.F. (2014). Epoxidation of olefins with molecular oxygen as the oxidant using gold catalysts supported on polyoxometalates. Green Chem..

[49] Huang Y., Liu Z., Gao G., Xiao G., Du A., Bottle S., Sarina S., Zhu H. (2017). Stable Copper Nanoparticle Photocatalysts for Selective Epoxidation of Alkenes with Visible Light. ACS Catal..

[50] Zwaschka G., Rondelli M., Krause M., Rötzer M.D., Hedhili M.N., Heiz U., Basset J.M., Schweinberger F.F., D’Elia V. (2018). Supported sub-nanometer Ta oxide clusters as model catalysts for the selective epoxidation of cyclooctene. New J. Chem..

[51] Schröder K., Join B., Amali A.J., Junge K., Ribas X., Costas M., Beller M. (2011). A Biomimetic Iron Catalyst for the Epoxidation of Olefins with Molecular Oxygen at Room Temperature. Angew. Chem. Int. Ed..

[52] Bouhlel E., Laszlo P., Levart M., Montaufier M.-T., Singh G.P. (1993). Epoxidation of olefins by molecular oxygen with clay-impregnated nickel catalysts. Tetrahedron Lett..

[53] Zhang J., Wei W.-J., Lu X., Yang H., Chen Z., Liao R.-Z., Yin G. (2017). Nonredox Metal Ions Promoted Olefin Epoxidation by Iron(II) Complexes with H_2_O_2_: DFT Calculations Reveal Multiple Channels for Oxygen Transfer. Inorg. Chem..

[54] Bregante D.T., Tan J.Z., Schultz R.L., Ayla E.Z., Potts D.S., Torres C., Flaherty D.W. (2020). Catalytic Consequences of Oxidant, Alkene, and Pore Structures on Alkene Epoxidations within Titanium Silicates. ACS Catal..

[55] Neves P., Nogueira L.S., Gomes A.C., Oliveira T.S.M., Lopes A.D., Valente A.A., Gonçalves I.S., Pillinger M. (2017). Chemistry and Catalytic Performance of Pyridyl-Benzimidazole Oxidomolybdenum(VI) Compounds in (Bio)Olefin Epoxidation. Eur. J. Inorg. Chem..

[56] Zwettler N., Schachner J.A., Belaj F., Mösch-Zanetti N.C. (2017). Hydrogen bond donor functionalized dioxido-molybdenum(VI) complexes as robust and highly efficient precatalysts for alkene epoxidation. Mol. Catal..

[57] Rossi-Fernández L., Dorn V., Radivoy G. (2021). A new and efficient methodology for olefin epoxidation catalyzed by supported cobalt nanoparticles. Beilstein J. Org. Chem..

[58] Bafti A., Razum M., Topić E., Agustin D., Pisk J., Vrdoljak V. (2021). Implication of oxidant activation on olefin epoxidation catalysed by Molybdenum catalysts with aroylhydrazonato ligands: Experimental and theoretical studies. Mol. Catal..

[59] Bhuiyan M.M., Mohammed M.L., Saha B. (2022). Greener and Efficient Epoxidation of 1,5-Hexadiene with *tert*-Butyl Hydroperoxide (TBHP) as an Oxidising Reagent in the Presence of Polybenzimidazole Supported Mo(VI) Catalyst. Reactions.

[60] Nunes M.S., Gomes A.C., Neves P., Mendes R.F., Almeida Paz F.A., Lopes A.D., Pillinger M., Gonçalves I.S., Valente A.A. (2023). Molybdenum(VI) complexes with ligands derived from 5-(2-pyridyl)-2H-tetrazole as catalysts for the epoxidation of olefins. Catal. Today.

[61] Samani M., Ardakani M.H., Sabet M. (2022). Efficient and selective oxidation of hydrocarbons with *tert*-butyl hydroperoxide catalyzed by oxidovanadium(IV) unsymmetrical Schiff base complex supported on γ-Fe_2_O_3_ magnetic nanoparticles. Res. Chem. Intermed..

[62] Fadaei Sarabi M., Bezaatpour A., Mahmoudi A. (2021). Anchoring of a terpyridine-based Mo(VI) complex on manganese ferrite as a recoverable catalyst for epoxidation of olefins under solvent-free conditions. J. Coord. Chem..

[63] Mirdarvatan V., Bahramian B., Khalaji A.D., Vaclavu T., Gómez-García C.J., Benmansour S., Triki S. (2023). A novel mixed azido/phenoxido bridged 1D CuII coordination polymer containing o-vanillin-based compartmental ligand: Synthesis, magnetism, phenoxazinone synthase and catalytic activity in epoxidation of alkenes. Polyhedron.

[64] Marreiros J., Diaz-Couce M., Ferreira M.J., Vaz P.D., Calhorda M.J., Nunes C.D. (2019). Synthesis and catalytic activity of Mo(II) complexes of α-diimines intercalated in layered double hydroxides. Inorganica Chim. Acta.

[65] Aigner M., Grosso-Giordano N.A., Okrut A., Zones S., Katz A. (2017). Epoxidation of 1-octene under harsh tail-end conditions in a flow reactor I: A comparative study of crystalline vs. amorphous catalysts. React. Chem. Eng..

[66] Blanco-Brieva G., Capel-Sanchez M.C., Campos-Martin J.M., Fierro J.L.G. (2007). Effect of precursor nature on the behavior of titanium-polysiloxane homogeneous catalysts in primary alkene epoxidation. J. Mol. Catal. A Chem..

[67] Bento A., Sanches A., Vaz P.D., Nunes C.D. (2016). Catalytic Application of Fe-doped MoO_2_ Tremella-Like Nanosheets. Top. Catal..

[68] Bento A., Sanches A., Medina E., Nunes C.D., Vaz P.D. (2015). MoO_2_ nanoparticles as highly efficient olefin epoxidation catalysts. Appl. Catal. A Gen..

[69] Naja I M., Abbasi A., Masteri-Farahani M. (2017). Preparation of MoO_3_/CuMoO_4_ nanoparticles as selective catalyst for olefin epoxidation. Sci. Iran..

[70] Zhang J., Zhang H., Liu L., Chen Z. (2022). The interaction of molybdenum and titanium in mesoporous materials for olefin epoxidation. React. Kinet. Mech. Catal..

[71] Martins A.M., Romão C.C., Abrantes M., Azevedo M.C., Cui J., Dias A.R., Duarte M.T., Lemos M.A., Lourenço T., Poli R. (2005). Mononuclear and Binuclear Cyclopentadienyl Oxo Molybdenum and Tungsten Complexes:  Syntheses and Applications in Olefin Epoxidation Catalysis. Organometallics.

[72] Huang J., Yuan L., Cai J., Liu Z. (2015). MoO_2_(acac)_2_ anchored on organic copolymer-inorganic hybrid zirconium phosphonate-phosphate functionalized by pyridines as highly efficient, reusable catalysts for alkene epoxidation. Microporous Mesoporous Mater..

[73] Masteri-Farahani M., Modarres M. (2017). Superiority of Activated Carbon versus MCM-41 for the Immobilization of Molybdenum Dithiocarbamate Complex as Heterogeneous Epoxidation Catalyst. ChemistrySelect.

[74] Nunes M.S., Gomes D.M., Gomes A.C., Neves P., Mendes R.F., Paz F.A.A., Lopes A.D., Valente A.A., Gonçalves I.S., Pillinger M. (2021). A 5-(2-Pyridyl)tetrazolate Complex of Molybdenum(VI), Its Structure, and Transformation to a Molybdenum Oxide-Based Hybrid Heterogeneous Catalyst for the Epoxidation of Olefins. Catalysts.

[75] Ambroziak K., Mbeleck R., He Y., Saha B., Sherrington D.C. (2009). Investigation of Batch Alkene Epoxidations Catalyzed by Polymer-Supported Mo(VI) Complexes. Ind. Eng. Chem. Res..

[76] Ambroziak K., Mbeleck R., Saha B., Sherrington D. (2010). Greener and Sustainable Method for Alkene Epoxidations by Polymer-Supported Mo(VI) Catalysts. Int. J. Chem. React. Eng..

[77] Liu X.-H., Yu H.-Y., Xue C., Zhou X.-T., Ji H.-B. (2020). Cyclohexene Promoted Efficient Biomimetic Oxidation of Alcohols to Carbonyl Compounds Catalyzed by Manganese Porphyrin under Mild Conditions. Chin. J. Chem..

[78] Zhou X.-T., Tang Q.-H., Ji H.-B. (2009). Remarkable enhancement of aerobic epoxidation reactivity for olefins catalyzed by μ-oxo-bisiron(III) porphyrins under ambient conditions. Tetrahedron Lett..

[79] Liu X.-H., Huang J.-Y., Tao L.-M., Yu H.-Y., Zhou X.-T., Xue C., Han Q., Zou W., Ji H.-B. (2022). Oxygen Atom Transfer Mechanism for Vanadium-Oxo Porphyrin Complexes Mediated Aerobic Olefin Epoxidation. Chin. J. Chem..

[80] Celano G., Šmejkalová D., Spaccini R., Piccolo A. (2008). Reduced Toxicity of Olive Mill Waste Waters by Oxidative Coupling with Biomimetic Catalysis. Environ. Sci. Technol..

[81] Zhang Y., Yang D., Li Y., Zhao X., Wang B., Qu J. (2019). Biomimetic catalytic oxidative coupling of thiols using thiolate-bridged dinuclear metal complexes containing iron in water under mild conditions. Catal. Sci. Technol..

[82] Esmelindro M.C., Oestreicher E.G., Márquez-Alvarez H., Dariva C., Egues S.M.S., Fernandes C., Bortoluzzi A.J., Drago V., Antunes O.A.C. (2005). Catalytic oxidation of cyclohexane by a binuclear Fe(III) complex biomimetic to methane monooxygenase. J. Inorg. Biochem..

[83] Luo W., Liu D., Sun J., Deng W., Sheng W., Liu Q., Guo C. (2014). Effects of Oxygen Transfer Limitation and Kinetic Control on Biomimetic Catalytic Oxidation of Toluene. Chin. J. Chem. Eng..

[84] Ibrahem I., Samec J.S.M., Bäckvall J.E., Córdova A. (2005). Enantioselective addition of aldehydes to amines via combined catalytic biomimetic oxidation and organocatalytic C-C bond formation. Tetrahedron Lett..

[85] Jana N.C., Patra M., Brandão P., Panja A. (2019). Synthesis, structure and diverse coordination chemistry of cobalt(III) complexes derived from a Schiff base ligand and their biomimetic catalytic oxidation of o-aminophenols. Polyhedron.

[86] Murahashi S.-I., Zhang D. (2008). Ruthenium catalyzed biomimetic oxidation in organic synthesis inspired by cytochrome P-450. Chem. Soc. Rev..

[87] Fang H., Wang M., Yi H., Zhang Y., Li X., Yan F., Zhang L. (2020). Electrostatic Assembly of Porphyrin-Functionalized Porous Membrane toward Biomimetic Photocatalytic Degradation Dyes. ACS Omega.

[88] Meng X., Yu C., Chen G., Zhao P. (2015). Heterogeneous biomimetic aerobic synthesis of 3-iodoimidazo [1,2-a]pyridines via CuO_x_/OMS-2-catalyzed tandem cyclization/iodination and their late-stage functionalization. Catal. Sci. Technol..

[89] Yan Z., Tian J., Wang K., Nigam K.D.P., Luo G. (2021). Microreaction processes for synthesis and utilization of epoxides: A review. Chem. Eng. Sci..

[90] Grigoropoulou G., Clark J.H., Elings J.A. (2003). Recent developments on the epoxidation of alkenes using hydrogen peroxide as an oxidant. Green Chem..

[91] Mandelli D., van Vliet M.C.A., Sheldon R.A., Schuchardt U. (2001). Alumina-catalyzed alkene epoxidation with hydrogen peroxide. Appl. Catal. A Gen..

[92] Lane B.S., Burgess K. (2003). Metal-Catalyzed Epoxidations of Alkenes with Hydrogen Peroxide. Chem. Rev..

[93] Stoica G., Santiago M., Jacobs P.A., Pérez-Ramírez J., Pescarmona P.P. (2009). Epoxidation catalysts derived from aluminium and gallium dawsonites. Appl. Catal. A Gen..

[94] Tse M.K., Klawonn M., Bhor S., Döbler C., Anilkumar G., Hugl H., Mägerlein W., Beller M. (2005). Convenient Method for Epoxidation of Alkenes Using Aqueous Hydrogen Peroxide. Org. Lett..

[95] Alvear M., Fortunato M.E., Russo V., Eränen K., Di Serio M., Lehtonen J., Rautiainen S., Murzin D., Salmi T. (2021). Continuous Liquid-Phase Epoxidation of Ethylene with Hydrogen Peroxide on a Titanium-Silicate Catalyst. Ind. Eng. Chem. Res..

[96] Yan W., Ramanathan A., Ghanta M., Subramaniam B. (2014). Towards highly selective ethylene epoxidation catalysts using hydrogen peroxide and tungsten or niobium-incorporated mesoporous silicate (KIT-6). Catal. Sci. Technol..

[97] Lu X., Zhou W.-J., Guan Y., Liebens A., Wu P. (2017). Enhancing ethylene epoxidation of a MWW-type titanosilicate/H_2_O_2_ catalytic system by fluorine implanting. Catal. Sci. Technol..

[98] Wang B., Zhu Y., Han H., Qin Q., Zhang Z., Zhu J. (2022). Preparation and Catalytic Performance in Propylene Epoxidation of Hydrophobic Hierarchical Porous TS-1 Zeolite. Catal. Lett..

[99] Li Y., Fan Q., Li Y., Feng X., Chai Y., Liu C. (2019). Seed-assisted synthesis of hierarchical nanosized TS-1 in a low-cost system for propylene epoxidation with H_2_O_2_. Appl. Surf. Sci..

[100] Maiti S.K., Ramanathan A., Subramaniam B. (2019). 110th Anniversary: Near-Total Epoxidation Selectivity and Hydrogen Peroxide Utilization with Nb-EISA Catalysts for Propylene Epoxidation. Ind. Eng. Chem. Res..

[101] Zuo Y., Liu M., Ma M., Song C., Guo X. (2017). Improved Catalytic Performance for 1-Butene Epoxidation over Titanium Silicalite-1 Extrudates by Using SBA-15 or Carborundum as Additives. Ind. Eng. Chem. Res..

[102] Egelske B.T., Xiong W., Zhou H., Monnier J.R. (2022). Effects of the method of active site characterization for determining structure-sensitivity in Ag-catalyzed ethylene epoxidation. J. Catal..

[103] Serafin J.G., Liu A.C., Seyedmonir S.R. (1998). Surface science and the silver-catalyzed epoxidation of ethylene: An industrial perspective. J. Mol. Catal. A Chem..

[104] Song Z., Yuan J., Cai Z., Lin D., Feng X., Sheng N., Liu Y., Chen X., Jin X., Chen D. (2020). Engineering three-layer core-shell S-1/TS-1@dendritic-SiO_2_ supported Au catalysts towards improved performance for propene epoxidation with H_2_ and O_2_. Green Energy Environ..

[105] Hu S., Li J., Wang Q., Yang W. (2022). Design and optimization of an integrated process for the purification of propylene oxide and the separation of propylene glycol by-product. Chin. J. Chem. Eng..

[106] Liu J., Ji X., Shi J., Wang L., Jian P., Yan X., Wang D. (2022). Experimental and theoretical investigation of the tuning of electronic structure in SnO_2_ via Co doping for enhanced styrene epoxidation catalysis. Catal. Sci. Technol..

[107] Tian S., Peng C., Dong J., Xu Q., Chen Z., Zhai D., Wang Y., Gu L., Hu P., Duan H. (2021). High-Loading Single-Atomic-Site Silver Catalysts with an Ag_1_-C_2_N_1_ Structure Showing Superior Performance for Epoxidation of Styrene. ACS Catal..

[108] Gupta R., Uslu H., Majumder S. (2022). Production of Styrene from Dehydrogenation of Ethylbenzene. Chem. Eng. Technol..

[109] Buijink J.K.F., van Vlaanderen J.J.M., Crocker M., Niele F.G.M. (2004). Propylene epoxidation over titanium-on-silica catalyst-the heart of the SMPO process. Catal. Today.

[110] De Vos D.E., Sels B.F., Jacobs P.A. (2003). Practical Heterogeneous Catalysts for Epoxide Production. Adv. Synth. Catal..

[111] Calvente R.M., Campos-Martin J.M., Fierro J.L.G. (2002). Effective homogeneous molybdenum catalyst for linear terminal alkenes epoxidation with organic hydroperoxide. Catal. Commun..

[112] Barrio L., Campos-Martín J.M., de Frutos M.P., Fierro J.L.G. (2008). Alkene Epoxidation with Ethylbenzene Hydroperoxides Using Molybdenum Heterogeneous Catalysts. Ind. Eng. Chem. Res..

[113] Levchuk I., Bhatnagar A., Sillanpää M. (2014). Overview of technologies for removal of methyl *tert*-butyl ether (MTBE) from water. Sci. Total Environ..

[114] Fadhli M., Khedher I., Fraile J.M. (2018). Enantioselective epoxidation of styrene with TBHP catalyzed by bis(oxazoline)–vanadyl–laponite materials. Catal. Commun..

[115] Zhang Y., Yang F., Gao R., Dai W.-L. (2019). Manganese-doped CeO_2_ nanocubes as highly efficient catalysts for styrene epoxidation with TBHP. Appl. Surf. Sci..

[116] Mohammed M.L., Saha B. (2022). Recent Advances in Greener and Energy Efficient Alkene Epoxidation Processes. Energies.

[117] Willms T., Kryk H., Hampel U. (2018). Partial Isobutane Oxidation to *tert*-Butyl Hydroperoxide in a Micro Reactor-Comparison of DTBP and Aqueous TBHP as Initiator. Chem. Ing. Tech..

[118] Wang Z., Balkus K.J. (2017). Liquid phase propylene oxidation with *tert*-butyl hydroperoxide over titanium containing wrinkled mesoporous silica. Catal. Commun..

[119] Chen D., Zhang X., Jiang H., Yuan X. (2020). Catalytic epoxidation of propylene over a Schiff-base molybdenum complex supported on a silanized mesostructured cellular foam. Res. Chem. Intermed..

[120] Chen M., Dai C., Yu G., Liu N., Xu R., Wang N., Chen B. (2022). Highly efficient absorption of methyl *tert*-butyl ether with ionic liquids. Sep. Purif. Technol..

[121] Ji B., Shao F., Hu G., Zheng S., Zhang Q., Xu Z. (2009). Adsorption of methyl *tert*-butyl ether (MTBE) from aqueous solution by porous polymeric adsorbents. J. Hazard. Mater..

[122] Nowacka A., Vismara R., Mercuri G., Moroni M., Palomino M., Domasevitch K.V., Di Nicola C., Pettinari C., Giambastiani G., Llabrés i Xamena F.X. (2020). Cobalt(II) Bipyrazolate Metal-Organic Frameworks as Heterogeneous Catalysts in Cumene Aerobic Oxidation: A Tag-Dependent Selectivity. Inorg. Chem..

[123] Nowacka A., Briantais P., Prestipino C., Llabrés i Xamena F.X. (2019). Selective Aerobic Oxidation of Cumene to Cumene Hydroperoxide over Mono- and Bimetallic Trimesate Metal-Organic Frameworks Prepared by a Facile “Green” Aqueous Synthesis. ACS Sustain. Chem. Eng..

[124] Li K.-T., Lin P.-H., Lin S.-W. (2006). Preparation of Ti/SiO_2_ catalysts by chemical vapor deposition method for olefin epoxidation with cumene hydroperoxide. Appl. Catal. A Gen..

[125] Xia Z., Li F., Xu L., Feng P. (2020). A stable and highly selective metalloporphyrin based framework for the catalytic oxidation of cyclohexene. Dalton Trans..

[126] Bach R.D., Canepa C., Winter J.E., Blanchette P.E. (1997). Mechanism of Acid-Catalyzed Epoxidation of Alkenes with Peroxy Acids. J. Org. Chem..

[127] Murphy A., Pace A., Stack T.D.P. (2004). Ligand and pH Influence on Manganese-Mediated Peracetic Acid Epoxidation of Terminal Olefins. Org. Lett..

[128] Wang Q., Gu Q., You S.-L. (2019). Enantioselective Carbonyl Catalysis Enabled by Chiral Aldehydes. Angew. Chem. Int. Ed..

[129] Li Y., Zhou X., Chen S., Luo R., Jiang J., Liang Z., Ji H. (2015). Direct aerobic liquid phase epoxidation of propylene catalyzed by Mn(iii) porphyrin under mild conditions: Evidence for the existence of both peroxide and Mn(iv)-oxo species from in situ characterizations. RSC Adv..

[130] Xu D., He Y., Liu X., Xiong C., Zhou X., Xue C., Ji H. (2021). *N*-Hydroxyphthalimide-Catalyzed Epoxidation of Inactive Aliphatic Olefins with Air at Room Temperature. Asian J. Org. Chem..

[131] Xiong C., He Y., Xu D., Liu X., Xue C., Zhou X., Ji H. (2022). Enhanced oxygen transfer over bifunctional Mo-based oxametallacycle catalyst for epoxidation of propylene. J. Colloid Interface Sci..

[132] Xiong C., Liang Y., Zhou X., Xue C., Ji H. (2023). Facile synthesis of a Mo-based TiO_2_ catalyst via a redox strategy for high value-added conversion of olefin. Fuel.

[133] Xu B., Deng M., Lin K., Wang Y., Lu X., Ma R., Fu Y., Zhu W. (2024). Ti-Beta zeotypes with open Ti(OSi)_3_OH sites for the efficient epoxidation of cyclohexene with H_2_O_2_. J. Catal..

[134] Zhang L., Mao S., Liu Y., Lu B., Wang Y., Li H., Wang Y. (2023). Tandem catalytic efficient olefin epoxidation with integrated production of nicotinamide derivatives. Chem Catal..

[135] Xiong C., Xue C., Yu X., He Y., Liang Y., Zhou X., Ji H. (2023). Tuning the olefin-VOCs epoxidation performance of ceria by mechanochemical loading of coinage metal. J. Hazard. Mater..

[136] Aoto H., Matsui K., Sakai Y., Kuchizi T., Sekiya H., Osada H., Yoshida T., Matsunaga S., Nomiya K. (2014). Zirconium(IV) and hafnium(IV)-containing polyoxometalates as oxidation precatalysts: Homogeneous catalytic epoxidation of cyclooctene by hydrogen peroxide. J. Mol. Catal. A Chem..

[137] Neves P., Gomes A.C., Paz F.A.A., Valente A.A., Gonçalves I.S., Pillinger M. (2017). Synthesis, structure and catalytic olefin epoxidation activity of a dinuclear oxo-bridged oxodiperoxomolybdenum(VI) complex containing coordinated 4,4′-bipyridinium. Mol. Catal..

[138] Priyarega S., Haribabu J., Karvembu R. (2022). Development of thiosemicarbazone-based transition metal complexes as homogeneous catalysts for various organic transformations. Inorganica Chim. Acta.

[139] Nogueira L.S., Neves P., Gomes A.C., Valente A.A., Pillinger M., Gonçalves I.S. (2017). Performance of a tetracarbonylmolybdenum(0) pyrazolylpyridine (pre)catalyst in olefin epoxidation and epoxide alcoholysis. J. Organomet. Chem..

[140] Gomes A.C., Bruno S.M., Tomé C., Valente A.A., Pillinger M., Abrantes M., Gonçalves I.S. (2013). Synthesis and characterization of C_p_Mo(CO)_3_(CH_2–p_C_6_H_4–_CO_2_CH_3_) and its inclusion compounds with methylated cyclodextrins. Applications in olefin epoxidation catalysis. J. Organomet. Chem..

[141] Sur A., Jernigan N.B., Powers D.C. (2022). Kinetic Probes of the Origin of Activity in MOF-Based C-H Oxidation Catalysis. ACS Catal..

[142] Karmadonova I.E., Zudin V.N., Kuznetsova N.I., Kuzhetsova L.I., Bal’zhinimaev B.S. (2020). Preparation of Ethylbenzene and Isopropylbenzene Hydroperoxides in the N-Hydroxyphthalimide-Fe(III) Homogeneous Catalytic System and Use of Solutions in the Epoxidation of Olefins. Catal. Ind..

[143] Chatterjee D., Mitra A. (1999). Olefin epoxidation catalysed by Schiff-base complexes of Mn and Ni in heterogenised-homogeneous systems. J. Mol. Catal. A Chem..

[144] Talzi E.P., Brylyakov K.P., Lyakin O.Y., Zima A.M., Soshnikov I.E. (2015). NMR and EPR spectroscopy applied to homogeneous catalysis. Kinet. Catal..

[145] Song Z., Liu X., Zhang H., Fang D., Ma X. (2021). Investigation of physicochemical properties for novel perrhenate ionic liquid and its catalytic application towards epoxidation of olefins. J. Chem. Sci..

[146] Escande V., Petit E., Garoux L., Boulanger C., Grison C. (2015). Switchable Alkene Epoxidation/Oxidative Cleavage with H_2_O_2_/NaHCO_3_: Efficient Heterogeneous Catalysis Derived from Biosourced Eco-Mn. ACS Sustain. Chem. Eng..

[147] Ren J., Wang L., Li P., Xing X., Wang H., Lv B. (2022). Ag supported on alumina for the epoxidation of 1-hexene with molecular oxygen: The effect of Ag^+^/Ag^0^. New J. Chem..

[148] Sun Q., Wang N., Yu J. (2021). Advances in Catalytic Applications of Zeolite-Supported Metal Catalysts. Adv. Mater..

[149] Liu Y., Wang S., Dai Z., Xiong Y. (2021). Cobalt Porphyrin-Cross-Linked Poly(Ionic Liquid)s as Efficient Heterogeneous Catalysts for Carbon Dioxide Conversion under Mild Conditions. Ind. Eng. Chem. Res..

[150] Wu Y., Wang H., Guo S., Zeng Y., Ding M. (2021). MOFs-induced high-amphiphilicity in hierarchical 3D reduced graphene oxide-based hydrogel. Appl. Surf. Sci..

[151] Mekrattanachai P., Liu J., Li Z., Cao C., Song W. (2018). Extremely low loading of Ru species on hydroxyapatite as an effective heterogeneous catalyst for olefin epoxidation. Chem. Commun..

[152] Sen R., Hazra D.K., Koner S., Helliwell M., Mukherjee M., Bhattacharjee A. (2010). Hydrothermal synthesis of dimeric lanthanide compounds: X-ray structure, magnetic study and heterogeneous catalytic epoxidation of olefins. Polyhedron.

[153] Nur H., Ikeda S., Ohtani B. (2001). Phase-Boundary Catalysis of Alkene Epoxidation with Aqueous Hydrogen Peroxide Using Amphiphilic Zeolite Particles Loaded with Titanium Oxide. J. Catal..

[154] Duff D.G., Ohrenberg A., Voelkening S., Boll M. (2004). A Screening Workflow for Synthesis and Testing of 10,000 Heterogeneous Catalysts per Day-Lessons Learned. Macromol. Rapid Commun..

[155] Zhang J., Jiang P., Shen Y., Zhang W., Bian G. (2016). Covalent anchoring of Mo(VI) Schiff base complex into SBA-15 as a novel heterogeneous catalyst for enhanced alkene epoxidation. J. Porous Mater..

[156] Fernandes C.I., Silva N.U., Vaz P.D., Nunes T.G., Nunes C.D. (2010). Bio-inspired Mo(II) complexes as active catalysts in homogeneous and heterogeneous olefin epoxidation. Appl. Catal. A Gen..

[157] Torres D., Lopez N., Illas F., Lambert R.M. (2005). Why Copper Is Intrinsically More Selective than Silver in Alkene Epoxidation:  Ethylene Oxidation on Cu(111) versus Ag(111). J. Am. Chem. Soc..

[158] Sha S., Yang H., Li J., Zhuang C., Gao S., Liu S. (2014). Co(II) coordinated metal-organic framework: An efficient catalyst for heterogeneous aerobic olefins epoxidation. Catal. Commun..

[159] Saha D., Gayen S., Koner S. (2018). Cu(II)/Cu(II)-Mg(II) containing pyridine-2,5-dicarboxylate frameworks: Synthesis, structural diversity, inter-conversion and heterogeneous catalytic epoxidation. Polyhedron.

[160] Baumes L.A., Serra J.M., Serna P., Corma A. (2006). Support Vector Machines for Predictive Modeling in Heterogeneous Catalysis:  A Comprehensive Introduction and Overfitting Investigation Based on Two Real Applications. J. Comb. Chem..

[161] Liu T.-T., Lin Z.-J., Shi P.-C., Ma T., Huang Y.-B., Cao R. (2015). A Metallosalen-based Porous Organic Polymer for Olefin Epoxidation. ChemCatChem.

[162] Yang F., Wang B., Zhou S., Long S., Liu X., Kong Y. (2017). Micropore-enriched CuO-based silica catalyst directly prepared by anionic template-induced method and its boosting catalytic activity in olefins epoxidation. Microporous Mesoporous Mater..

[163] He S., Liu X., Zhao H., Zhu Y., Zhang F. (2015). Zirconium phenylphosphonate-anchored methyltrioxorhenium as novel heterogeneous catalyst for epoxidation of cyclohexene. J. Colloid Interface Sci..

[164] Baumes L.A., Blansché A., Serna P., Tchougang A., Lachiche N., Collet P., Corma A. (2009). Using Genetic Programming for an Advanced Performance Assessment of Industrially Relevant Heterogeneous Catalysts. Mater. Manuf. Process..

[165] Umsa J., Mingqiao Z., Xinzhi C., Zhangfa T. (2017). Polyoxometalate-nano Gold Hybrid Mesostructured Catalyst for Green Cyclohexene Epoxidation. Curr. Org. Chem..

[166] He X., He Q., Deng Y., Peng M., Chen H., Zhang Y., Yao S., Zhang M., Xiao D., Ma D. (2019). A versatile route to fabricate single atom catalysts with high chemoselectivity and regioselectivity in hydrogenation. Nat. Commun..

[167] Jiang J., Chen H.-Y., Zhou X.-T., Chen Y.-J., Xue C., Ji H.-B. (2020). Biomimetic Aerobic Epoxidation of Alkenes Catalyzed by Cobalt Porphyrin under Ambient Conditions in the Presence of Sunflower Seeds Oil as a Co-Substrate. ACS Omega.

[168] He Q., Zhang Y., Xiao H., He X., Zhou X., Ji H. (2019). Facile Synthesis of Metalloporphyrins-Ba^2+^ Composites as Recyclable and Efficient Catalysts for Olefins Epoxidation Reactions. Chem. Res. Chin. Univ..

[169] Ge Y., Su X., Li G., Yang Y.-F., She Y. (2023). Oxidative functionalization of naphthalene derivatives catalyzed by Mn(III)-porphyrins. Tetrahedron Lett..

[170] Zhang Y., Cao C., She Y., Yang Y.-F., Houk K.N. (2023). Molecular Dynamics of Iron Porphyrin-Catalyzed C-H Hydroxylation of Ethylbenzene. J. Am. Chem. Soc..

[171] Yuan R., Wei Y., Xue Z., Wang A., Zhang J., Xu H., Zhao L. (2023). Effects of support material and electrolyte on a triphenylamine substituted cobalt porphyrin catalytic oxygen reduction reaction. Colloids Surf. A Physicochem. Eng. Asp..

[172] Deeba R., Collard A., Rollin C., Molton F., Chardon-Noblat S., Costentin C. (2023). Controlled Potential Electrolysis: Transition from Fast to Slow Regimes in Homogeneous Molecular Catalysis. Application to the Electroreduction of CO_2_ Catalyzed by Iron Porphyrin. ChemElectroChem.

[173] Wu F., Jiang F., Yang J., Dai W., Lan D., Shen J., Fang Z. (2023). Investigation of Molecular Mechanism of Cobalt Porphyrin Catalyzed CO_2_ Electrochemical Reduction in Ionic Liquid by In-Situ SERS. Molecules.

[174] Ding D., Chen X., Su X., She Y.-B., Yang Y.-F. (2023). Computational insights into the dual reactivity of 1,2,3,4-tetrazole: A metalloporphyrin-catalyzed click reaction and denitrogenative annulation. Org. Chem. Front..

[175] Fan J., Wang Y., Hu X., Liu Y., Che C.-M. (2023). Iron porphyrin-catalysed C(sp^3^)–H amination with alkyl azides for the synthesis of complex nitrogen-containing compounds. Org. Chem. Front..

[176] Murata S., Kurosawa M., Fujisawa T. (2023). Efficient synthesis of carbon-14 labeled metabolites of the strobilurin fungicide mandestrobin using biomimetic iron-porphyrin catalyzed oxidation. J. Label. Compd. Radiopharm..

[177] Cao Y.-C., Shi L.-L., Li M., You B., Liao R.-Z. (2023). Deciphering the Selectivity of the Electrochemical CO_2_ Reduction to CO by a Cobalt Porphyrin Catalyst in Neutral Aqueous Solution: Insights from DFT Calculations. ChemistryOpen.

[178] Zhou X.-T., Wang L.-L., Li Y., Ji H.-B. (2022). Liquid-phase epoxidation of propylene with molecular oxygen by chloride manganese meso-tetraphenylporphyrins. Chin. J. Chem. Eng..

[179] Zhou X.-T., Yu H.-Y., Li Y., Xue C., Ji H.-B. (2020). Cerium(IV) Sulfate as a Cocatalyst for Promoting the Direct Epoxidation of Propylene by Ruthenium Porphyrin with Molecular Oxygen. Ind. Eng. Chem. Res..

[180] Mahmoudi H., Bagherzadeh M., Ataie S., Kia R., Moravej S.H., Zare M., Raithby P.R., Ferlin F., Vaccaro L. (2020). Synthesis and X-ray Crystal Structure of a Molybdenum(VI) Schiff Base Complex: Design of a New Catalytic System for Sustainable Olefin Epoxidation. Inorganica Chim. Acta.

[181] Adam M.S.S., Ahmed M.S.M., El-Hady O.M., Shaaban S. (2020). Bis-dioxomolybdenum (VI) oxalyldihydrazone complexes: Synthesis, characterization, DFT studies, catalytic epoxidation potential, molecular modeling and biological evaluations. Appl. Organomet. Chem..

[182] Mandal M., Nagaraju V., Karunakar G.V., Sarma B., Borah B.J., Bania K.K. (2015). Electronic, Conjugation, and Confinement Effects on Structure, Redox, and Catalytic Behavior of Oxido-Vanadium(IV) and -(V) Chiral Schiff Base Complexes. J. Phys. Chem. C.

[183] Lazar A., Thiel W.R., Singh A.P. (2014). Synthesis and characterization of 3-[N,N′-bis-3-(salicylidenamino)ethyltriamine] Mo(vi)O2@SBA-15: A highly stable and reusable catalyst for epoxidation and sulfoxidation reactions. RSC Adv..

[184] Mirzaee M., Bahramian B., Shahraki M., Moghadam H., Mirzaee A. (2018). Molybdenum Containing Catalysts Grafted on Functionalized Hydrous Zirconia Nano-particles for Epoxidation of Alkenes. Catal. Lett..

[185] Ramanathan A., Wu J.-F., Maheswari R., Hu Y., Subramaniam B. (2017). Synthesis of molybdenum-incorporated mesoporous silicates by evaporation-induced self-assembly: Insights into surface oxide species and corresponding olefin metathesis activity. Microporous Mesoporous Mater..

[186] Bagherzadeh M., Zare M., Amani V., Ellern A., Keith Woo L. (2013). Dioxo and oxo-peroxo molybdenum(VI) complexes bearing salicylidene 2-picoloyl hydrazone: Structures and catalytic performances. Polyhedron.

[187] Neves P., Lysenko A.B., Gomes A.C., Pillinger M., Gonçalves I.S., Valente A.A. (2017). Behavior of Triazolylmolybdenum(VI) Oxide Hybrids as Oxidation Catalysts with Hydrogen Peroxide. Catal. Lett..

[188] Schachner J.A., Mösch-Zanetti N.C., Peuronen A., Lehtonen A. (2017). Dioxidomolybdenum(VI) and tungsten(VI) complexes with tetradentate amino bisphenolates as catalysts for epoxidation. Polyhedron.

[189] Lysenko A.B., Senchyk G.A., Domasevitch K.V., Kobalz M., Krautscheid H., Cichos J., Karbowiak M., Neves P., Valente A.A., Gonçalves I.S. (2017). Triazolyl, Imidazolyl, and Carboxylic Acid Moieties in the Design of Molybdenum Trioxide Hybrids: Photophysical and Catalytic Behavior. Inorg. Chem..

[190] Deubel D.V., Frenking G., Gisdakis P., Herrmann W.A., Rösch N., Sundermeyer J. (2004). Olefin Epoxidation with Inorganic Peroxides. Solutions to Four Long-Standing Controversies on the Mechanism of Oxygen Transfer. Acc. Chem. Res..

[191] Liu X., Gu Q., Zhang Y., Xu X., Wang H., Sun Z., Cao L., Sun Q., Xu L., Wang L. (2023). Atomically Thick Oxide Overcoating Stimulates Low-Temperature Reactive Metal–Support Interactions for Enhanced Catalysis. J. Am. Chem. Soc..

[192] Ghorbanloo M., Heydari A., Yahiro H. (2018). Ag-nanoparticle embedded p(AA) hydrogel as an efficient green heterogeneous Nano-catalyst for oxidation and reduction of organic compounds. Appl. Organomet. Chem..

[193] Chakraborty T., Chakraborty A., Maity S., Das D., Chattopadhyay T. (2019). Conglomerated system of Ag nanoparticles decorated Al_2_O_3_ supported cobalt and copper complexes with enhanced catalytic activity for oxidation reactions. Mol. Catal..

[194] Hoseinzade K., Mousavi-Mashhadi S.A., Shiri A. (2022). An efficient and green one-pot synthesis of tetrahydrobenzo[a]xanthenes, 1,8-dioxo-octahydroxanthenes and dibenzo[a,j]xanthenes by Fe_3_O_4_@Agar-Ag as nanocatalyst. Mol. Divers..

[195] Khan M., Janjua N.K., Khan S., Qazi I., Ali S., Saad Algarni T. (2021). Electro-Oxidation of Ammonia at Novel Ag_2_O−PrO_2_/γ-Al_2_O_3_ Catalysts. Coatings.

[196] Zhao Z., Xu H., Feng Z., Zhang Y., Cui M., Cao D., Cheng D. (2020). Design of High-Performance Co-Based Alloy Nanocatalysts for the Oxygen Reduction Reaction. Chem.-A Eur. J..

[197] Mullangi D., Chakraborty D., Pradeep A., Koshti V., Vinod C.P., Panja S., Nair S., Vaidhyanathan R. (2018). Heterogenous Catalysts: Highly Stable COF-Supported Co/Co(OH)_2_ Nanoparticles Heterogeneous Catalyst for Reduction of Nitrile/Nitro Compounds under Mild Conditions (Small 37/2018). Small.

[198] Gholinejad M., Zareh F., Najera C. (2018). Iron oxide modified with pyridyl-triazole ligand for stabilization of gold nanoparticles: An efficient heterogeneous catalyst for A3 coupling reaction in water. Appl. Organomet. Chem..

[199] Liu Y., Li W., Zhao G., Qin G., Li Y., Liu Y. (2021). Self-driven microstructural evolution of Au@Pd core–shell nanoparticles for greatly enhanced catalytic performance during methanol electrooxidation. Nanoscale.

[200] Yazdani H., Pardis S., Loni M., Bazgir A. (2020). Gold nanoparticle as a Lewis acid catalyst in 1,3-dipolar cycloaddition reaction. Catal. Commun..

[201] Zhao J., Yuan H., Yang G., Liu Y., Qin X., Chen Z., Weng-Chon C., Zhou L., Fang S. (2022). AuPt bimetallic nanoalloys supported on SBA-15: A superior catalyst for quinoline selective hydrogenation in water. Nano Res..

[202] Chakraborty D., Nandi S., Mullangi D., Haldar S., Vinod C.P., Vaidhyanathan R. (2019). Cu/Cu_2_O Nanoparticles Supported on a Phenol–Pyridyl COF as a Heterogeneous Catalyst for the Synthesis of Unsymmetrical Diynes via Glaser-Hay Coupling. ACS Appl. Mater. Interfaces.

[203] Zhu G., Jin Y., Ge M. (2022). Simple and green method for preparing copper nanoparticles supported on carbonized cotton as a heterogeneous Fenton-like catalyst. Colloids Surf. A Physicochem. Eng. Asp..

[204] Dehghani F., Sardarian A.R., Esmaeilpour M. (2013). Salen complex of Cu(II) supported on superparamagnetic Fe3O4@SiO2 nanoparticles: An efficient and recyclable catalyst for synthesis of 1- and 5-substituted 1H-tetrazoles. J. Organomet. Chem..

[205] Xu Q., Guo C., Li B., Zhang Z., Qiu Y., Tian S., Zheng L., Gu L., Yan W., Wang D. (2022). Al^3+^ Dopants Induced Mg^2+^ Vacancies Stabilizing Single-Atom Cu Catalyst for Efficient Free-Radical Hydrophosphinylation of Alkenes. J. Am. Chem. Soc..

[206] Geesi M.H., Ouerghi O., Elsanousi A., Kaiba A., Riadi Y. (2021). Ultrasound-Assisted Preparation of Cu-Doped TiO_2_ Nanoparticles as a Nanocatalyst for Sonochemical Synthesis of Pyridopyrimidines. Polycycl. Aromat. Compd..

[207] Liu K., Qin R., Zheng N. (2021). Insights into the Interfacial Effects in Heterogeneous Metal Nanocatalysts toward Selective Hydrogenation. J. Am. Chem. Soc..

[208] Imran M., Yousaf A.B., Zhou X., Jiang Y.-F., Yuan C.-Z., Zeb A., Jiang N., Xu A.-W. (2017). Pd/TiO Nanocatalyst with Strong Metal-Support Interaction for Highly Efficient Durable Heterogeneous Hydrogenation. J. Phys. Chem. C.

[209] Das M.R., Hussain N., Duarah R., Sharma N., Sarmah P., Thakur A., Bhattacharjee P., Bora U., Boukherroub R. (2024). Metal nanoparticles decorated two-dimensional nanosheets as heterogeneous catalysts for coupling reactions. Catal. Rev..

[210] Bisio C., Carniato F., Palumbo C., Safronyuk S.L., Starodub M.F., Katsev A.M., Marchese L., Guidotti M. (2016). Nanosized inorganic metal oxides as heterogeneous catalysts for the degradation of chemical warfare agents. Catal. Today.

[211] Dhakshinamoorthy A., Navalon S., Alvaro M., Garcia H. (2012). Metal Nanoparticles as Heterogeneous Fenton Catalysts. ChemSusChem.

[212] Gawande M.B., Goswami A., Felpin F.-X., Asefa T., Huang X., Silva R., Zou X., Zboril R., Varma R.S. (2016). Cu and Cu-Based Nanoparticles: Synthesis and Applications in Catalysis. Chem. Rev..

[213] Miyaji T., Wu P., Tatsumi T. (2001). Selective oxidation of propylene to propylene oxide over Ti-MCM-41 supporting metal nitrate. Catal. Today.

[214] Sharma A.S., Sharma V.S., Kaur H., Varma R.S. (2020). Supported heterogeneous nanocatalysts in sustainable, selective and eco-friendly epoxidation of olefins. Green Chem..

[215] Tyablikov I., Romanovsky B. (2016). A heterogeneous organocatalyst for olefin epoxidation. Catal. Today.

[216] van Hoof A.J.F., Filot I.A.W., Friedrich H., Hensen E.J.M. (2018). Reversible Restructuring of Silver Particles during Ethylene Epoxidation. ACS Catal..

[217] Lei Y., Mehmood F., Lee S., Greeley J., Lee B., Seifert S., Winans R.E., Elam J.W., Meyer R.J., Redfern P.C. (2010). Increased Silver Activity for Direct Propylene Epoxidation via Subnanometer Size Effects. Science.

[218] Kim K.J., Rath M.K., Kwak H.H., Kim H.J., Han J.W., Hong S.-T., Lee K.T. (2019). A Highly Active and Redox-Stable SrGdNi_0.2_Mn_0.8_O_4±δ_ Anode with in Situ Exsolution of Nanocatalysts. ACS Catal..

[219] Yang H., Li G., Jiang G., Zhang Z., Hao Z. (2023). Heterogeneous selective oxidation over supported metal catalysts: From nanoparticles to single atoms. Appl. Catal. B Environ..

[220] Yang H., Ma C., Li Y., Wang J., Zhang X., Wang G., Qiao N., Sun Y., Cheng J., Hao Z. (2018). Synthesis, characterization and evaluations of the Ag/ZSM-5 for ethylene oxidation at room temperature: Investigating the effect of water and deactivation. Chem. Eng. J..

[221] Yang H., Ma C., Wang G., Sun Y., Cheng J., Zhang Z., Zhang X., Hao Z. (2018). Fluorine-enhanced Pt/ZSM-5 catalysts for low-temperature oxidation of ethylene. Catal. Sci. Technol..

[222] Yu Y., Li Y., Zhang X., Deng H., He H., Li Y. (2015). Promotion Effect of H_2_ on Ethanol Oxidation and NO_x_ Reduction with Ethanol over Ag/Al_2_O_3_ Catalyst. Environ. Sci. Technol..

[223] Yang H., Ma C., Zhang X., Li Y., Cheng J., Hao Z. (2018). Understanding the Active Sites of Ag/Zeolites and Deactivation Mechanism of Ethylene Catalytic Oxidation at Room Temperature. ACS Catal..

[224] Christopher P., Linic S. (2008). Engineering Selectivity in Heterogeneous Catalysis: Ag Nanowires as Selective Ethylene Epoxidation Catalysts. J. Am. Chem. Soc..

[225] Ramirez A., Hueso J.L., Suarez H., Mallada R., Ibarra A., Irusta S., Santamaria J. (2016). A Nanoarchitecture Based on Silver and Copper Oxide with an Exceptional Response in the Chlorine-Promoted Epoxidation of Ethylene. Angew. Chem. Int. Ed..

[226] Jing X., Wang H., Chen H., Huang J., Li Q., Sun D. (2014). Biosynthesized Ag/α-Al_2_O_3_ catalyst for ethylene epoxidation: The influence of silver precursors. RSC Adv..

[227] Ghosh S., Acharyya S.S., Tiwari R., Sarkar B., Singha R.K., Pendem C., Sasaki T., Bal R. (2014). Selective Oxidation of Propylene to Propylene Oxide over Silver-Supported Tungsten Oxide Nanostructure with Molecular Oxygen. ACS Catal..

[228] Lu G., Zuo X. (1999). Epoxidation of propylene by air over modified silver catalyst. Catal. Lett..

[229] Lee E.J., Lee J., Seo Y.-J., Lee J.W., Ro Y., Yi J., Song I.K. (2017). Direct epoxidation of propylene to propylene oxide with molecular oxygen over Ag-Mo-W/ZrO_2_ catalysts. Catal. Commun..

[230] Haruta M., Kobayashi T., Sano H., Yamada N. (1987). Novel Gold Catalysts for the Oxidation of Carbon Monoxide at a Temperature far Below 0 °C. Chem. Lett..

[231] Hutchings G.J. (1985). Vapor phase hydrochlorination of acetylene: Correlation of catalytic activity of supported metal chloride catalysts. J. Catal..

[232] Haruta M. (2011). Spiers Memorial Lecture Role of perimeter interfaces in catalysis by gold nanoparticles. Faraday Discuss..

[233] Haruta M., Uphade B.S., Tsubota S., Miyamoto A. (1998). Selective oxidation of propylene over gold deposited on titanium-based oxides. Res. Chem. Intermed..

[234] Uphade B.S., Akita T., Nakamura T., Haruta M. (2002). Vapor-Phase Epoxidation of Propene Using H_2_ and O_2_ over Au/Ti–MCM-48. J. Catal..

[235] Kapoor M.P., Sinha A.K., Seelan S., Inagaki S., Tsubota S., Yoshida H., Haruta M. (2002). Hydrophobicity induced vapor-phase oxidation of propene over gold supported on titanium incorporated hybrid mesoporous silsesquioxane. Chem. Commun..

[236] Chen S., Li D., Cao T., Huang W. (2020). Size-Dependent Structures and Catalytic Performances of Au/TiO_2_-{001} Catalysts for Propene Epoxidation. J. Phys. Chem. C.

[237] Uphade B.S., Okumura M., Tsubota S., Haruta M. (2000). Effect of physical mixing of CsCl with Au/Ti-MCM-41 on the gas-phase epoxidation of propene using H_2_ and O_2_: Drastic depression of H_2_ consumption. Appl. Catal. A Gen..

[238] Zhang Z., Tang Y., Du W., Xu J., Wang Q., Song N., Qian G., Duan X., Zhou X. (2022). Engineering gold impregnated uncalcined TS-1 to boost catalytic formation of propylene oxide. Appl. Catal. B: Environ..

[239] Pulido A., Boronat M., Corma A. (2012). Propene Epoxidation with H_2_/H_2_O/O_2_ Mixtures Over Gold Atoms Supported on Defective Graphene: A Theoretical Study. J. Phys. Chem. C.

[240] Lu J., Luo M., Lei H., Bao X., Li C. (2002). Epoxidation of Propylene on NaCl-Modified VCe1-x Cux Oxide Catalysts with Direct Molecular Oxygen as the Oxidant. J. Catal..

[241] Monnier J.R., Hartley G.W. (2001). Comparison of Cu and Ag Catalysts for Epoxidation of Higher Olefins. J. Catal..

[242] Hua Q., Cao T., Gu X.-K., Lu J., Jiang Z., Pan X., Luo L., Li W.-X., Huang W. (2014). Crystal-Plane-Controlled Selectivity of Cu_2_O Catalysts in Propylene Oxidation with Molecular Oxygen. Angew. Chem. Int. Ed..

[243] Long W., Zhai Q., He J., Zhang Q., Deng W., Wang Y. (2012). Significant Synergistic Effect between Supported Ruthenium and Copper Oxides for Propylene Epoxidation by Oxygen. ChemPlusChem.

[244] Seubsai A., Uppala C., Tiencharoenwong P., Chukeaw T., Chareonpanich M., Zohour B., Noon D., Senkan S. (2018). High Stability of Ruthenium-Copper-Based Catalysts for Epoxidation of Propylene. Catal. Lett..

[245] Lei J., Dai J., Tan K.B., Huang J., Zhan G., Li Q. (2021). Insight into the Effect of Copper Substitution on the Catalytic Performance of LaCoO_3_-Based Catalysts for Direct Epoxidation of Propylene with Molecular Oxygen. ACS Sustain. Chem. Eng..

[246] He J., Zhai Q., Zhang Q., Deng W., Wang Y. (2013). Active site and reaction mechanism for the epoxidation of propylene by oxygen over CuO_x_/SiO_2_ catalysts with and without Cs^+^ modification. J. Catal..

[247] Song Z., Mimura N., Bravo-Suárez J.J., Akita T., Tsubota S., Oyama S.T. (2007). Gas-phase epoxidation of propylene through radicals generated by silica-supported molybdenum oxide. Appl. Catal. A Gen..

[248] Murata K., Liu Y., Mimura N., Inaba M. (2003). Direct vapor phase oxidation of propylene by molecular oxygen over MCM-41 or MCM-22 based catalysts. Catal. Commun..

[249] Yan W., Ramanathan A., Patel P.D., Maiti S.K., Laird B.B., Thompson W.H., Subramaniam B. (2016). Mechanistic insights for enhancing activity and stability of Nb-incorporated silicates for selective ethylene epoxidation. J. Catal..

[250] Munnik P., de Jongh P.E., de Jong K.P. (2015). Recent Developments in the Synthesis of Supported Catalysts. Chem. Rev..

[251] Jin H., Zhou K., Zhang R., Cui H., Yu Y., Cui P., Song W., Cao C. (2023). Regulating the electronic structure through charge redistribution in dense single-atom catalysts for enhanced alkene epoxidation. Nat. Commun..

[252] Zhao Q., Xu D., Wang L., Cui S., Liu Q., Han X., Wang Z., Ning H., Wu M. (2025). A core-shell confinement strategy towards single-atom Fe-N/S-C bifunctional catalyst for selective nitroarene reduction and olefin epoxidation. J. Alloys Compd..

[253] Niatouri A.D., Yadollahi B. (2023). A novel POM/LDH/GO nanocomposite as highly efficient heterogeneous catalyst in green epoxidation of alkenes with hydrogen peroxide. Catal. Commun..

[254] Yuan J., Meng H., Li Y., Liu Y., Wang Y., Liu J., Zhou Z. (2024). An ionic liquid in Core-shell structure: Halogen-free, metal-free bifunctional catalyst for olefin epoxidation and CO_2_ cycloaddition. J. CO_2_ Util..

[255] Ozbek M.O., Onal I., van Santen R.A. (2012). Effect of Surface and Oxygen Coverage on Ethylene Epoxidation. Top. Catal..

[256] Linic S., Barteau M.A. (2002). Formation of a Stable Surface Oxametallacycle that Produces Ethylene Oxide. J. Am. Chem. Soc..

[257] Linic S., Barteau M.A. (2003). Control of Ethylene Epoxidation Selectivity by Surface Oxametallacycles. J. Am. Chem. Soc..

[258] Linic S., Piao H., Adib K., Barteau M.A. (2004). Ethylene Epoxidation on Ag: Identification of the Crucial Surface Intermediate by Experimental and Theoretical Investigation of its Electronic Structure. Angew. Chem. Int. Ed..

[259] Christopher P., Linic S. (2010). Shape and Size-Specific Chemistry of Ag Nanostructures in Catalytic Ethylene Epoxidation. ChemCatChem.

[260] Özbek M.O., van Santen R.A. (2013). The Mechanism of Ethylene Epoxidation Catalysis. Catal. Lett..

[261] Ozbek M.O., Onal I., Van Santen R.A. (2011). Ethylene epoxidation catalyzed by chlorine-promoted silver oxide. J. Phys. Condens. Matter.

[262] Ozbek M.O., Onal I., van Santen R.A. (2011). Why silver is the unique catalyst for ethylene epoxidation. J. Catal..

[263] Clerici M.G., Ingallina P. (1993). Epoxidation of Lower Olefins with Hydrogen Peroxide and Titanium Silicalite. J. Catal..

[264] Xiong C., Liu H., Zhou J., Xu D., Liang Y., Zhou X., Xue C., Ji H. (2023). High selective epoxidation of 2-methylpropene over a Mo-based oxametallacycle reinforced nano composite. Nano Res..

[265] Li J., Zhou X., Ao J., Gao J., Wang A., Shen Z., Gu Y., Zhou J., Chen Y. (2024). P450DA Monooxygenase-Catalyzed Chemoselective and Enantiodivergent Epoxidation of Unactivated Alkenes. ACS Catal..

[266] Wu H., Xu Y., Ao J., Guo P., Xu Y., Huang Z., Zhang L. (2024). Electrochemical epoxidation of alkene with high faradaic efficiencies using water as an oxygen source. Green Chem..

[267] Kim S.S., Hong S., Surendran A.K., Roy A., Malik D.D., Chun D., Kim S., Kim Y., Lee Y.M., Lee Y.H. (2025). Electrochemically Driven Selective Olefin Epoxidation by Cobalt-TAML Catalyst. J. Am. Chem. Soc..

[268] Gadolini S., Kerber R.N., Seljamäe-Green R.T., Tong W., Farràs P., Corbos E.C. (2024). Covalently Anchored Molecular Catalyst onto a Graphitic Carbon Nitride Surface for Photocatalytic Epoxidation of Olefins. ACS Catal..

